# Intra-patient stability of tumor mutational burden from tissue biopsies at different time points in advanced cancers

**DOI:** 10.1186/s13073-021-00979-8

**Published:** 2021-10-12

**Authors:** Timothy V. Pham, Aaron M. Goodman, Smruthy Sivakumar, Garrett Frampton, Razelle Kurzrock

**Affiliations:** 1 Center for Personalized Cancer Therapy, University of California San Diego (UCSD), 3855 Health Sciences Drive, La Jolla, CA 92037 USA; 2 Division of Blood and Marrow Transplantation, UCSD, 3855 Health Sciences Drive, MC-0960, La Jolla, CA 92093 USA; 3 Foundation Medicine, Inc, 150 Second Street, Cambridge, MA 02141 USA

**Keywords:** Tumor mutational burden, Immunotherapy, Immunotherapy effect on TMB, TMB over time, TMB over treatment

## Abstract

**Background:**

Tumor mutational burden (TMB) may be a predictive biomarker of immune checkpoint inhibitor (ICI) responsiveness. Genomic landscape heterogeneity is a well-established cancer feature. Molecular characteristics may differ even within the same tumor specimen and undoubtedly evolve with time. However, the degree to which TMB differs between tumor biopsies within the same patient has not been established.

**Methods:**

We curated data on 202 patients enrolled in the PREDICT study (NCT02478931), seen at the University of California San Diego (UCSD), who had 404 tissue biopsies for TMB (two per patient, mean of 722 days between biopsies) to assess difference in TMB before and after treatment in a pan-cancer cohort. We also performed an orthogonal analysis of 2872 paired pan-solid tumor biopsies in the Foundation Medicine database to examine difference in TMB between first and last biopsies.

**Results:**

The mean (95% CI) TMB difference between samples was 0.583 [− 0.900–2.064] (*p* = 0.15). Pearson correlation showed a flat line for time elapsed between biopsies versus TMB change indicating no correlation (*R*^2^ = 0.0001; *p* = 0.8778). However, in 55 patients who received ICIs, there was an increase in TMB (before versus after mean mutations/megabase [range] 12.50 [range, 0.00–98.31] versus 14.14 [range, 0.00–100.0], *p* = 0.025). Analysis of 2872 paired pan-solid tumor biopsies in the Foundation Medicine database also indicated largely stable TMB patterns; TMB increases were only observed in specific tumors (e.g., breast, colorectal, glioma) within certain time intervals.

**Conclusions:**

Overall, our results suggest that tissue TMB remains stable with time, though specific therapies such as immunotherapy may correlate with an increase in TMB.

**Trial registration:**

NCT02478931, registered June 23, 2015.

**Supplementary Information:**

The online version contains supplementary material available at 10.1186/s13073-021-00979-8.

## Background

Tumor mutational burden (TMB) is currently emerging as a predictive biomarker of response to checkpoint blockade immunotherapy as noted by the recent Food and Drug Administration approval of the anti-programmed cell death protein (PD-1) antibody pembrolizumab for solid cancers with TMB ≥ 10 mutations/Mb [1–4], though there is still controversy regarding its value due to its variable distribution depending on cancer type [5–8], sequencing methodology, and lack of standards for the definition of “high,” “medium,” or “low” TMB, and the consequent lack of harmonized clinical validation [9]. Some studies have shown TMB to be a more robust biomarker for its correlation with outcome than simply programmed death ligand 1 (PD-L1) expression alone [10], which is one of the current National Comprehensive Cancer Network (NCCN) criteria for prescribing checkpoint blockade immunotherapy [11].

Checkpoint blockade immunotherapies target a tumor immune evasion mechanism by interfering with the interaction between tumor-expressed PD-L1 and PD-1 expressed on cytotoxic T-cells. Logically, to be a valid attack vector, a T cell must first be able to recognize the cancer cell as foreign through the peptide antigens the tumor cell presents on its surface, themselves a product of the tumor’s genome, which may be mutated due to factors such as exposure to environmental carcinogens like tobacco smoke and ultraviolet radiation, as well as dysregulated endogenous mechanisms such as apolipoprotein B mRNA-editing enzyme and catalytic polypeptide-like (APOBEC) enzyme-mediated *kataegis* [12–15]. The more mutations (and hence the higher the TMB), presumably the better the chance for immune recognition [13].

There has been scant analysis regarding if and how a tumor’s TMB changes over the course of disease and treatment. However, Gerlinger et. al. [16] showed that tumors are tremendously heterogenous even within the same patient [16]. Further, Riaz et al. [17] reported that TMB decreases in melanoma when immunotherapy treatment is successful and that certain subclonal populations within the tumor are eliminated due to the action of tumor infiltrating lymphocytes (TIL) activated by the checkpoint inhibitor nivolumab, changing the tumor’s composition and therefore post-treatment immune microenvironment [17].

Our aim was to determine if TMB changed over time in patients with repeated biopsies. We found that TMB across cancer types remained stable between biopsies and any differences in TMB did not correlate with time elapsed between biopsies. However, within specific tumor types and/or with specific therapies such as immunotherapy, an increase in TMB with time was observed.

## Methods

### Patient and sample demographics

We examined clinical data, specifically diagnosis and oncology drug treatments with associated dates and outcomes, sex, and dates of birth and last follow-up or death, from 225 University of California San Diego (UCSD) patients enrolled in the UCSD PREDICT study (Trial Registration NCT02478931, https://clinicaltrials.gov/ct2/show/NCT02478931) who had ≥ 2 biopsies that underwent next-generation sequencing (NGS). This was a non-therapeutic, correlative study of personalized medicine with retrospective and prospective components. Patient medical records are examined for results of molecular profiling obtained through standard of care testing to help understand, in a descriptive fashion, how well molecular testing might predict response to therapy. Patient outcome parameters including, but not limited to, tumor response, time to treatment failure, patient survival, and toxicity were analyzed, as well as pharmacodynamic (PD) and pharmacokinetic (PK) data when available. This study also included research-related testing of tissue, blood, or urine specimens via a variety of simple or advanced techniques such as molecular, proteomic, and metabolic analyses for biomarker discovery or for PK and PD parameters.

Eligibility and inclusion/exclusion critereria were: All patients with a diagnosis of cancer or cancer-related referred to a UCSD Health System facility, Eisenhower Medical Center (EMC), and Rady Children’s Hospital - San Diego (RCHSD) were eligible. Patients or their legal guardians must be willing and able to provide written informed consent to participate in the prospective part of the study unless the patient has been lost to clinical follow-up.

The data was curated from patient charts stored on the UCSD Moores Cancer Center Electronic Medical Records system. Data on the patients’ somatic mutations, TMB, and microsatellite status were obtained from the NGS reports provided by Foundation Medicine. From there, only patients whose NGS reports were from a tumor of the same histology with at least two valid, numerical TMBs, who had valid diagnosis data and distinct test dates were selected, leaving 202 patients in total (Additional file 1: Fig. S1, CONSORT Diagram). The difference in TMB between the earliest and latest biopsies, in chronological order, was quantified for further analysis. The 202 UCSD patients were then dichotomized into groups representing drug type exposure (immunotherapy and non-immunotherapy), and the TMB difference between the groups was tested for statistical significance. This study was performed in accordance with UCSD IRB guidelines for the PREDICT protocol (NCT02478931, https://clinicaltrials.gov/ct2/show/NCT02478931) and for any investigational treatments or procedures for which patients gave consent. Research followed the Declaration of Helsinki guidelines. Waiver of consent was permitted by protocol and UCSD IRB for non-identifying retrospective data collection and data analysis. The de-identified UCSD patient data with all variables used in the analysis is available in Additional file 2: Table S2. The study protocol for the PREDICT study is available in Additional file 3: PREDICT Study Protocol. The consent form for the PREDICT study is available in Additional file 3: PREDICT Consent Form.

In addition to the dataset above, we assessed paired tumor biopsy samples from 2872 patients (Additional file 2: Table S1, Additional file 2: Table S3) who underwent targeted comprehensive genomic profiling as part of routine clinical care at Foundation Medicine, as described previously in Frampton et al [18]. For patients with more than two available samples, we included only the first and last sample for paired TMB assessments. We further examined tumor types with at least 50 pairs and binned the collection time difference between the biopsies into the following groups: ≤ 365 days, 366 to 1095 days, and > 1095 days. Approval for the Foundation Medicine study including a waiver of informed consent and a Health Insurance Portability and Accountability Act (HIPAA) waiver of authorization was obtained from the Western Institutional Review Board (Protocol No. 20152817). The de-identified Foundation Medicine patient data with all variables used in the previously described analysis is available in Additional file 2: Table S3.

### TMB evaluation

In both the UCSD and Foundation Medicine cohorts, mutations in each sample were identified using hybrid-capture-based NGS on formalin-fixed paraffin embedded (FFPE) tumor samples by Foundation Medicine’s clinical laboratory improvement amendments (CLIA)-certified lab, specifically with the FoundationOne, FoundationOne®CDx, or FoundationOne Heme panels containing 182, 236, 315, or 406 genes depending on the date of sequencing and test type, as previously described in [18] (Foundation Medicine, Cambridge, MA, USA; http://www.foundationone.com/). These different panel types were used in order to maximize the amount of available data due to the multi-year and multi-diagnosis nature of the study. On average, the sequencing depth for > 99% of exons was greater than 100x, for a total depth of coverage of at least 250x covering 1.2 Megabases (Mb). The numerical TMB in mutations/Mb was assessed by extrapolating the number of mutations captured by the panel to the whole genome with a validated algorithm [19]. Germline and characterized oncogenic alterations were not included. The biopsied samples were trichotomized into three groups of low (≤ 5 mutations/Mb), intermediate (between 6 and 19 mutations/Mb, inclusive), and high (≥ 20 mutations/Mb) TMB [20].

### Statistical analysis

A Wilcoxon signed-rank test (non-parametric paired test) was used to compare the earlier TMB with the later TMB due to the linked nature of the before-and-after comparison. Statistical significance for the effects of immunotherapy on TMB change was determined using a 2-tailed Mann-Whitney *U* test. Correlation between time elapsed between biopsies and measured TMB difference was assessed with linear regression and the *R*^2^ of the Pearson correlation was reported to show strength of correlation. *p* values of ≤ 0.05 were considered statistically significant. These statistical tests were performed using GraphPad Prism® version 6.01 (San Diego, CA, USA).

## Results

**Fig. 1:**
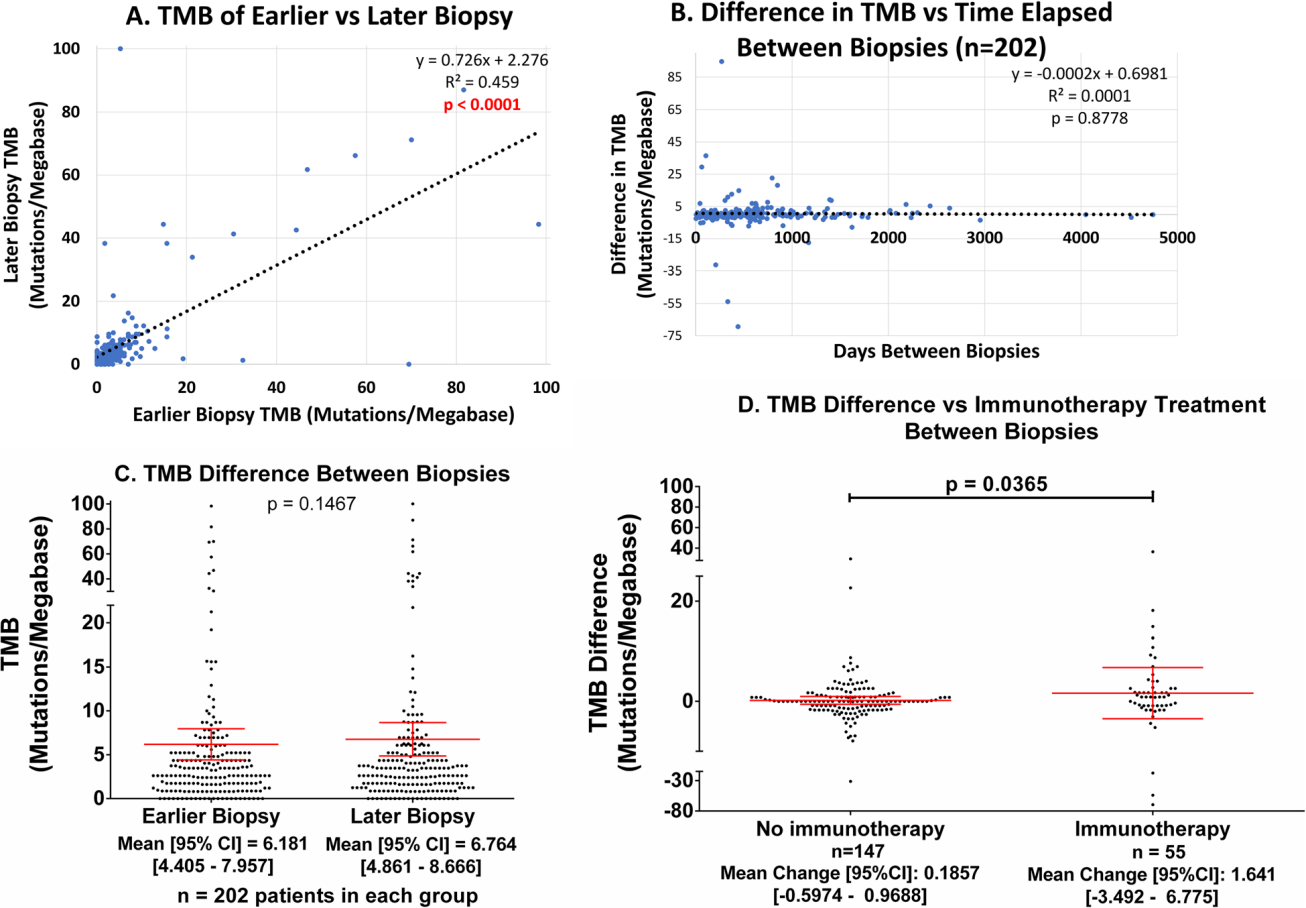
UCSD TMB differences. All patients with valid UCSD TMB data (*n* = 202) were considered for this figure. Exposure or treatment is defined as having received agent between the initial and final biopsies, regardless of dose or duration, as per electronic medical records. **A** The TMB of the earlier and later biopsies are somewhat correlated with a significant, almost unity, slope (*p* < 0.0001), and a Pearson *R*^2^ = 0.459. This indicates there is no difference between early and later TMBs. **B** TMB difference does not correlate with time elapsed between biopsies (Pearson correlation *R*^2^ = 0.0001). The slope of the line of best fit is also not significantly greater than zero (*p* = 0.8778). **C** There is no significant different between TMBs measured at different times as determined by the Wilcoxon matched pairs signed rank test. Red lines are mean (center) ± 95% confidence interval (CI) for each group. The average TMB of the earlier biopsy was 6.181 [4.405–7.957] mutations/Mb versus 6.764 [4.861–8.666] mutations/Mb for the later biopsy (*p* = 0.1467). **D** TMB difference (increase) with time in immunotherapy-treated patients is greater than in those who did not receive immunotherapy (immunotherapy response not considered). All patients with valid TMB data considered. Red lines are mean (center) ± 95% CI for each group. Patients who were treated with immunotherapy between biopsies had a mean ± 95% CI TMB change of 1.641 [− 3.492–6.775] mutations/Mb whereas those who were not had a mean ± 95% CI TMB change of 0.1857 [− 0.5974–0.9688] mutations/Mb (*p* = 0.0365). Drugs received included the following: ipilimumab, nivolumab, pembrolizumab, atezolizumab, avelumab, durvalumab, and cemiplimab

In the UCSD clinically curated cohort of 202 patients with 404 tissue biopsies for TMB (two biopsies per patient), the average TMB change over the course of treatment, regardless of agent, was 0.583 mutations/Mb (95% CI − 0.900–2.064 mutations/mb), with an average time between biopsies of 722 days (95% CI 621–821 days) (Table 1). There was no significant difference in TMB between the earlier and later biopsies (*p* = 0.15) (Fig. 1c). The slope of the linear regression between earlier and later biopsies is significant (*p* < 0.0001) with a moderate Pearson correlation of *R*^2^ = 0.459 (Fig. 1a). Additional file 1: Fig. S2a and S2b also show that this correlation is stable if we analyze TMB 0 to 20 mutations/mb or if we log transform the TMB to attenuate the influence of outliers.

**Table 1:** Demographic/biopsy data of UCSD patient cohort with evaluable TMBs for multiple biopsies and clinical data

Factor	Item			Value [95% CI]
	Average time between biopsies (days)			722 [621–821]
	Average TMB difference (mutations/Mb)			0.583 [− 0.900–2.064]
	Age at first biopsy (years)			57.36 [55.22–59.50]
**Gender**	Men			106 (52.48%)
	Women			96 (47.52%)
**Diagnoses**	Colorectal adenocarcinoma			29 (14.36%)
	Lung adenocarcinoma			20 (9.901%)
	Breast invasive ductal adenocarcinoma			10 (4.950%)
	Metastatic (any primary)			99 (49.00%)
**Biopsy sites**	**First biopsy**		**Last biopsy**	
	Lung	22 (10.89%)	Liver	33 (16.34%)
	Colon	19 (9.406%)	Lung	20 (9.901%)
	Soft Tissue	16 (7.921%)	Lymph Node	16 (7.921%)
	Bone Marrow	11 (5.446%)	Bone Marrow	14 (6.931%)
	Liver	11 (5.446%)	Colon	12 (5.941%)
	Others	123 (60.89%)	Others	107 (52.97%)
**Immunotherapy (checkpoint)**	Number receiving immunotherapy between biopsies			55 (27.23%)
	Number NOT receiving immunotherapy			147 (72.77%)
**TMB measured**	High at first biopsy (≥ 20 mutations/Mb)			10 (4.950%)
	Intermediate at first biopsy (5 < TMB ≤ 19 mutations/Mb)			49 (24.26%)
	Low at first biopsy (≤ 5 mutations/Mb)			143 (70.79%)
	High at second biopsy (≥ 20 mutations/Mb)			13 (6.436%)
	Intermediate at second biopsy (5 < TMB ≤ 19 mutations/Mb)			50 (24.75%)
	Low at second biopsy (≤ 5 mutations/Mb)			139 (68.81%)

Patients in the UCSD cohort (*N* = 202) tend to be older and are roughly evenly distributed between men and women. Almost half (49%) of the patients have metastatic cancer. 55 of them (27.23%) received immunotherapy between their first and last biopsies, on average 722 days apart

Data derived from medical record; if tobacco or alcohol exposure not known, then patients not counted

There was also no correlation (Pearson *R*^2^ = 0.0001) between time elapsed between biopsy and TMB difference (Fig. 1b), and the slope of the line of best fit for time elapsed between biopsy and TMB difference is also not significantly different than zero (*p* = 0.8778) (Fig. 1b).

**Fig. 2:**
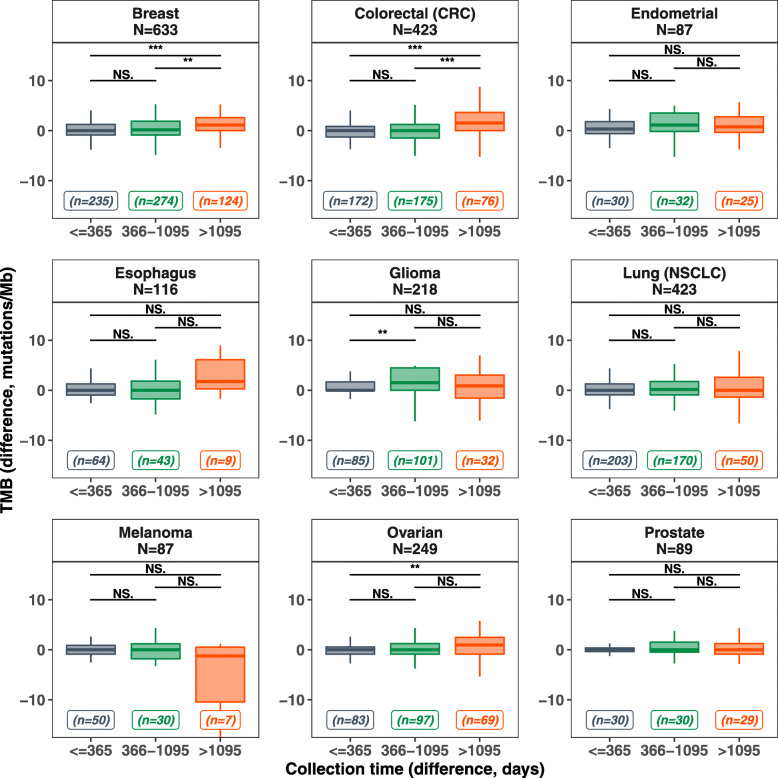
Foundation Medicine TMB differences in categorical elapsed time. A total of 2872 paired tissue biopsy samples from the Foundation Medicine database were studied for patterns of TMB change based on time between tests. Tumor types with at least 50 pairs were considered for this figure. Each panel represents a specific tumor type with the total number of assessed samples denoted by “*N*.” The collection time differences were binned into three categories: ≤ 365 days, 366 to 1095 days, and > 1095 days. Boxplots of the TMB change were plotted for each bin of collection time difference, with the total number of pairs in each bin denoted by “*n*.” In each boxplot: the horizontal line represents the median, the box represents the interquartile range (IQR) and the whiskers represent extremes of the data (capped at 1.5xIQR). Wilcoxon test for statistical difference for each pairwise comparison was performed; the *p* value is presented based on different thresholds (*0.05, **0.01, ***0.001, NS.: not significant). The TMB difference (*y*-axis) is capped to be between − 15 and 15 for better visualization; however, statistical analysis was performed on all available samples. Individual data points for TMB change, colored by the categorical time between biopsy, are available in Additional file 1: Fig. S4. See Additional file 2: Table S1 for detailed data and for false discovery rate adjusted *p* value for multiple comparisons

In the 55 patients who received checkpoint blockade immunotherapy between biopsies, the mean (range) TMB in mutations/mb before immunotherapy was 12.50 (range 0.00 to 98.31) versus 14.14 (range 0.00 to 100.0) after immunotherapy (*p* = 0.025) (Table 2). Patients exposed to immunotherapy between biopsies (*N* = 55) had an average TMB change of 1.641 mutations/Mb (95% CI − 3.492–6.775) mutations/mb whereas those without had an average TMB change of − 0.166 (95% CI − 1.524–1.193) mutations/mb, a significant difference (*p* = 0.037) (Fig. 1d, Table 2). If the 14 patients who had breast, colon, and ovarian cancer or gliomas (the diseases that showed some increase in TMB with time in the larger Foundation Medicine dataset (Fig. 2)) were eliminated from this analysis, there was still a significant increase in TMB with time in the immunotherapy-treated 41 patients without these diagnoses (*p* = 0.0202)

**Table 2:** Immunotherapy exposure between biopsies and effects on TMB in UCSD cohort (*N* = 202)

Drug	Mean TMB [95% CI] (mutations/Mb)			Number receiving/total
	Before	After	***p*** value	
**Immunotherapy**	12.50 [6.565–18.43]	14.14 [7.960–20.32]	0.0252	55/202 (27.23%)
	**Mean [95% CI] TMB difference (mutations/Mb), with immunotherapy** **(*n*** **= 55)**	**Mean [95% CI] TMB difference (mutations/Mb), no immunotherapy** **(*n*** **= 147)**	***p*** **value**	
	1.641 [− 3.492–6.775]	0.186 [− 0.597–0.969]	0.0365	

Difference in TMB between first and last biopsies were compared for patients exposed to medication, in any amount, between biopsy dates versus difference in TMB between first and last biopsies for all patients who did not receive the specified medication(s) between the biopsy dates. Only the 202 patients with valid TMB data were considered. The results show a significant difference (*p* < 0.05) in TMB difference and overall TMB before and after immunotherapy use

Of the 2872 pairs of TMB samples from the same patient in the Foundation Medicine database, we examined disease groups with at least 50 pairs: breast cancer (*N* = 633 pairs), colorectal (*N* = 423), non-small cell lung cancer (*N* = 423), ovarian cancer (*N* = 249), glioma (*N* = 218), esophageal cancer (*N* = 116), melanoma (*N* = 89), prostate cancer (*N* = 87), and endometrial cancer (*N* = 87) (Fig. 2, Additional file 2: Table S1, Additional file 1: Fig. S3, Additional file 1: Fig. S4). In the 2872 paired samples, the median collection time between samples was 448 days (range, 0 to 5444 days). The average change of TMB over time was 1.58 mutations/mb (95% confidence interval [CI], 1.04 to 2.12 mutations/mb). Overall, there was no correlation between time elapsed and TMB change (Pearson correlation coefficient = 0.041, *p* = 0.03) (Additional file 1: Fig. S3A, B). However, certain tumor types showed significant difference in TMB change between the different binned collection time differences (Fig. 2). Breast and colorectal tumors showed statistically significant differences between the collection time bins (≤ 365 days) and (> 1095 days) as well as between the collection time bins (366-1095 days) and (> 1095 days), whereas gliomas showed a statistically significant difference relatively early, between collection time bins (≤ 365 days) and (366–1095 days). TMB values at earlier and later biopsies were also well-correlated with one another, with linear regression slopes approaching 1 and significantly non-zero (all *p* < 1 × 10^−4^) when each histology group was analyzed separately (Additional file 1: Fig. S3C), and for all paired biopsy samples in the Foundation Medicine, samples were analyzed together (Additional file 1: Fig. S3D). When the TMB values were logarithmically transformed, the results remained consistent, meaning that outlier TMB values did not substantially influence the results (Additional file 1: Fig. S3E, F).

## Discussion

TMB is a quantification of molecular alterations, often expressed in mutations per megabase and determined via genomic sequencing methods such as whole genome or exome sequencing [3] or extrapolation from sequencing part of the genome. TMB may reflect to what extent a tumor’s cells will present mutated antigens that the host’s immune system can recognize and bind to, in order to be primed for checkpoint blockade immunotherapy. Much has been written about TMB’s potential as a positive predictor of response pre-treatment [3, 21, 22] in cohorts of specific cancers such as lung [22], melanoma, and glioblastoma [23], as well as in pan-cancer cohorts [1, 24], though not all studies agree and the predictive power of TMB for immunotherapy response remains a matter of debate. Still, the reason behind checkpoint blockade’s resounding success in melanoma may be because melanoma is characterized by a high mutational burden [14]. High TMB is correlated with disabling mutations in mismatch repair genes such as *MSH2*, *MSH6*, *MLH1*, *POLE*, PMS2, and *POLD1* [23], which is logical since mutations are a product of DNA repair deficiencies; further, mismatch repair defects are themselves another potential marker of immunotherapy response [25]. Higher TMB is associated with higher neo-antigen load, perhaps explaining the immune responsiveness.

Molecular heterogeneity, even in the same tumor sample as well as those emanating from the same primary, but separated spatially and temporally, has been well described and confounds genomic-based treatments [16]. However, to date, to our knowledge, whether or not heterogeneity in TMB exists as well has not been investigated. Our study shows that, in 2872 pairs of TMB samples from the same patient in the Foundation Medicine database, with a median collection time between samples of 448 days, there was no correlation between time elapsed and TMB change. Importantly, however, TMB changes were observed in specific tumor types within the Foundation Medicine cohort: breast and colorectal tumors and gliomas showed statistically significant TMB increases between certain time bins, with gliomas showing these significant increases in TMB relatively early. Similarly, in 202 clinically curated UCSD patients from the PREDICT study with two tissue biopsies separated by a mean of 722 days, there was no material change in TMB across tumor types. Further, the time interval between biopsies had virtually no effect on the TMB level. However, patients that had received checkpoint blockade between biopsies did show a statistically significant increase in TMB. The latter results differ from those previously published by Riaz et al. [17] who reported that TMB decreases in melanoma when immunotherapy treatment is effective. Of note, we did not sort our patients by response, in part because only 55 immunotherapy-treated patients were available. The fact that non-responders were included in our cohort (and the majority of patients do not respond to immunotherapy) may easily explain the differing results between our study and that of Riaz et al. [17]. Even so, these observations require validation in by other investigators, as the numerical increase between time points is small. Of note, the TMB changes observed in specific tumor types within the large Foundation Medicine cohort may potentially, in part, be attributed to therapy as well. For example, the observed TMB increase in gliomas could be reflective of treatment with temozolomide, a previously known mechanism [26]. However, the lack of treatment information for this cohort makes it unfeasible to test for such patterns. Finally, there were individual patients with large positive or negative swings in TMB; these individuals were disproportionately represented in the immunotherapy-treated cohort.

Our observations have several limitations, including their retrospective nature and the fact that, in the UCSD clinically curated database, because of the heterogeneity of treatments in this group of individuals, the confounding influence of therapies other than immunotherapy was not addressed, and requires a prospective study. Single cell resolution sequencing data, which would have been necessary to distinguish subclonal characteristics within each patient such as TMB, mutation, and expression patterns, and could have provided biological explanation for our observations, was not available to us. Moreover, binning of time periods was based on availability of samples, and even larger sample sizes with different bins might produce additional information. Finally, the large Foundation Medicine dataset did not have clinical therapy curated (though this was curated for the UCSD dataset) and could be biased by selection based on which patients had sequencing performed in the first place, and which patients survived and had a second test done. The lack of information about therapies used, biopsy sites, and lack of subclonal information also limits the translational utility of the data set. Despite these limitations, both the UCSD and Foundation Medicine cohorts show that TMB is largely invariant over time.

## Conclusions

In summary, we find that TMB remains stable between tumor biopsies regardless of time interval between sampling. However, in specific tumor types such as breast, colorectal cancer, and gliomas, there may be increases in TMB with time. Furthermore, individual therapies such as immunotherapy may be associated with an overall increase in TMB.

## Supplementary Information

**Additional file 1: Figure S1.** CONSORT Diagram for UCSD clinically curated cohort. **Figure S2.** Additional correlation analysis between earlier and later TMB for UCSD clinically curated cohort. **Figure S3.** Linear Regression Analysis of Paired Biopsies in the Foundation Medicine Cohort. **Figure S4.** Difference in TMB from paired biopsies in Foundation Medicine Cohort

**Additional file 2: **Foundation Medicine and UCSD Cohort Characteristics. **Table S1.** Patterns of TMB difference based on time between biopsies in the Foundation Medicine cohort. **Table S2.** Patients with paired tissue biopsy samples in the UCSD cohort. **Table S3.** Patients with paired tissue biopsy samples in the Foundation Medicine cohort

**Additional file 3:.** PREDICT Study Protocol and Consent Form. Study Protocol. Consent Form

## Data Availability

De-identified data of the variables used for statistical analysis of the 202 patients from the UCSD PREDICT cohort are available in Additional file 2: Table S2. De-identified, consented data for the 2872 patients in the Foundation Medicine cohort analyzed in the current study are included in Additional file 2: Table S3. All patients/participants in this study did not consent to release of raw sequence data for confidentiality or privacy purposes/in compliance with the ethics approvals obtained for this study.

## Abbreviations

TMB: Tumor mutational burden
*N*: Number
CI: Confidence interval
PD-1: Programmed cell death protein 1
PD-L1: Programmed death ligand 1
NCCN: National Comprehensive Cancer Network
APOBEC: Apolipoprotein B mRNA-editing enzyme, catalytic polypeptide-like
TIL: Tumor infiltrating lymphocyte
NGS: Next-generation sequencing
FFPE: Formalin-fixed paraffin embedded
CLIA: Clinical laboratory improvement amendments
Mb: Megabase
