# Supplementary material for: Intra-patient stability of tumor mutational burden from tissue biopsies at different time points in advanced cancers
Source: Genome Med. 2021 Oct 12;13:159. doi: 10.1186/s13073-021-00979-8 (PMC8513181; doi:10.1186/s13073-021-00979-8)
Supplement: Supplementary file 1 — Additional file 1: Figure S1. CONSORT Diagram for UCSD clinically curated cohort. Figure S2. Additional correlation analysis between earlier and later TMB for UCSD clinically curated cohort. Figure S3. Linear Regression Analysis of Paired Biopsies in the Foundation Medicine Cohort. Figure S4. Difference in TMB from paired biopsies in Foundation Medicine Cohort [file 13073_2021_979_MOESM1_ESM.docx]

# Additional File 1: Supplementary Figures

Protocol-eligible patients with tumors tested with FoundationOne, FoundationOne Dx, or HemeComplete Tests with two TMB estimates on different samples collected at different times, but tumors of same histology (n=203 patients, 406 TMBs) *.

Patient with invalid/unclear diagnosis information (n=1 patient, 2 TMBs)

Patients with valid/clear diagnosis information (n=202 patients, 404 TMBs)

* If patients had more than two biopsies for TMB, we only considered those from the earliest and latest dated collection times.

**Figure S1: CONSORT Diagram for UCSD clinically curated cohort.**

203 patients enrolled in the PREDICT study with at least 2 Tumor Mutational Burden data points, as assessed by Foundation Medicine NGS testing, were identified in the UCSD Moores Cancer database. Of these patients, one was excluded for having invalid, non-informative diagnosis information, leaving 202 patients and 404 numerical TMB data points total. This data is available in **Additional File 2: Table S1**.

##
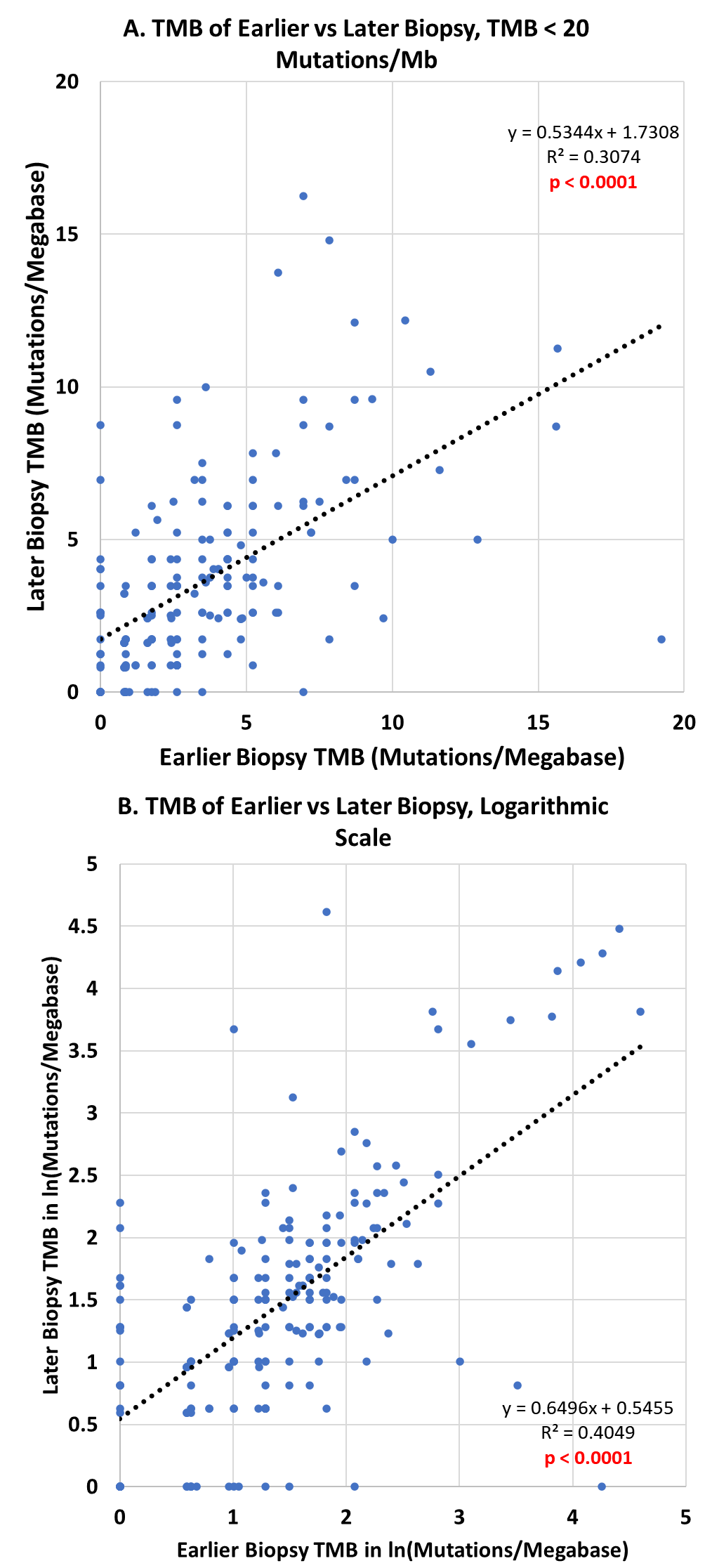
Figure S2: Additional correlation analysis between earlier and later TMB for UCSD clinically curated cohort

Both these sub-analyses of the TMB correlation data in Figure 2A indicate that outliers had a limited effect on the correlation between earlier and later biopsies.

A. The correlation between the TMB of the earlier and later biopsies whose TMB were both between 0 – 20 Mutations/Mb was weaker than in the full data set with R^2^ = 0.3074 although the slope was still significantly different from zero (p < 0.0001).

B. Logarithmically transforming the earlier and later TMB values in order to moderate the influence of outliers also results in a slightly weaker correlation (R^2^ = 0.4059). The slope of the line of best fit was also remained significantly different from zero (p < 0.0001).

##
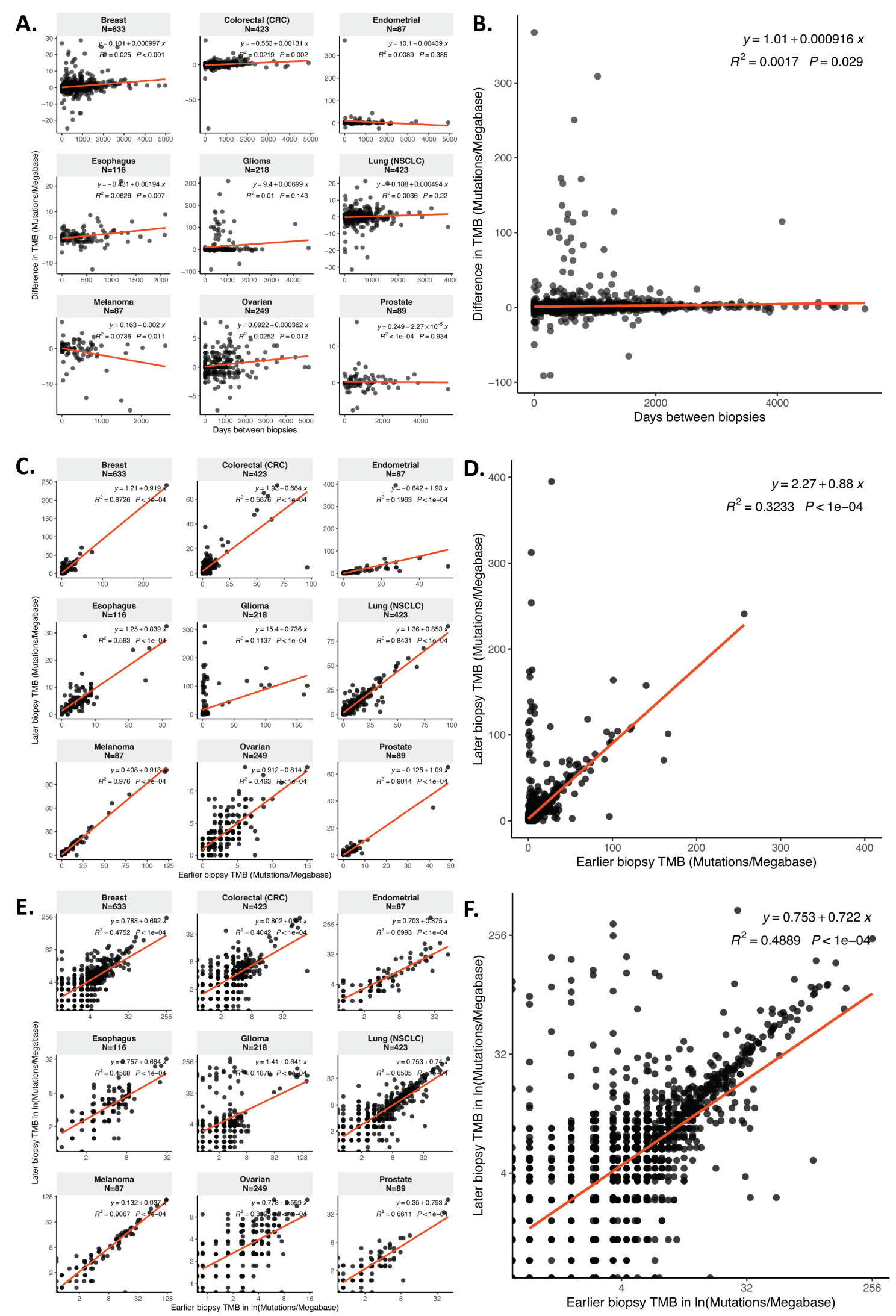
 Figure S3: Linear Regression Analysis of Paired Biopsies in the Foundation Medicine Cohort

Linear regression analysis of first and last TMB and TMB difference vs time shows no change between biopsies.

A. In the 9 diagnosis groups of the Foundation Medicine data set, detailed in Figure 1, TMB change was not well-correlated with time between biopsy (all R^2^ < 0.1). Although the deviation of the line of best fit’s slope was significant in breast, colorectal, esophagus, glioma, melanoma, and ovarian cancers (p < 0.05), the slope was relatively small on the order of magnitude of 0.001 (thousandths) Mutations/Mb/day.

B. When the TMB change of all samples in the Foundation Medicine data set were plotted against days between biopsies, the R^2^ remained low at 0.0017, indicating a poor correlation.

C. The TMB values of the earlier and later biopsies were well correlated with each other in the Foundation Medicine data set when each histological group was analyzed separately. In all cases, the slope of the linear regression line was approximately 1 and significantly different from zero (all p < 1 x 10^-4^).

D. This correlation between first and last biopsy TMB remained when all Foundation Medicine samples were pooled together.

E. Taking the natural logarithm of the first and last TMB values preserves the correlation when each diagnosis group is analyzed separately, indicating that outliers have a limited effect on the linear regression.

F. In the full Foundation Medicine data set, the logarithmic transforms of the TMB values remain correlated (R^2^ = 0.4889) with one another.


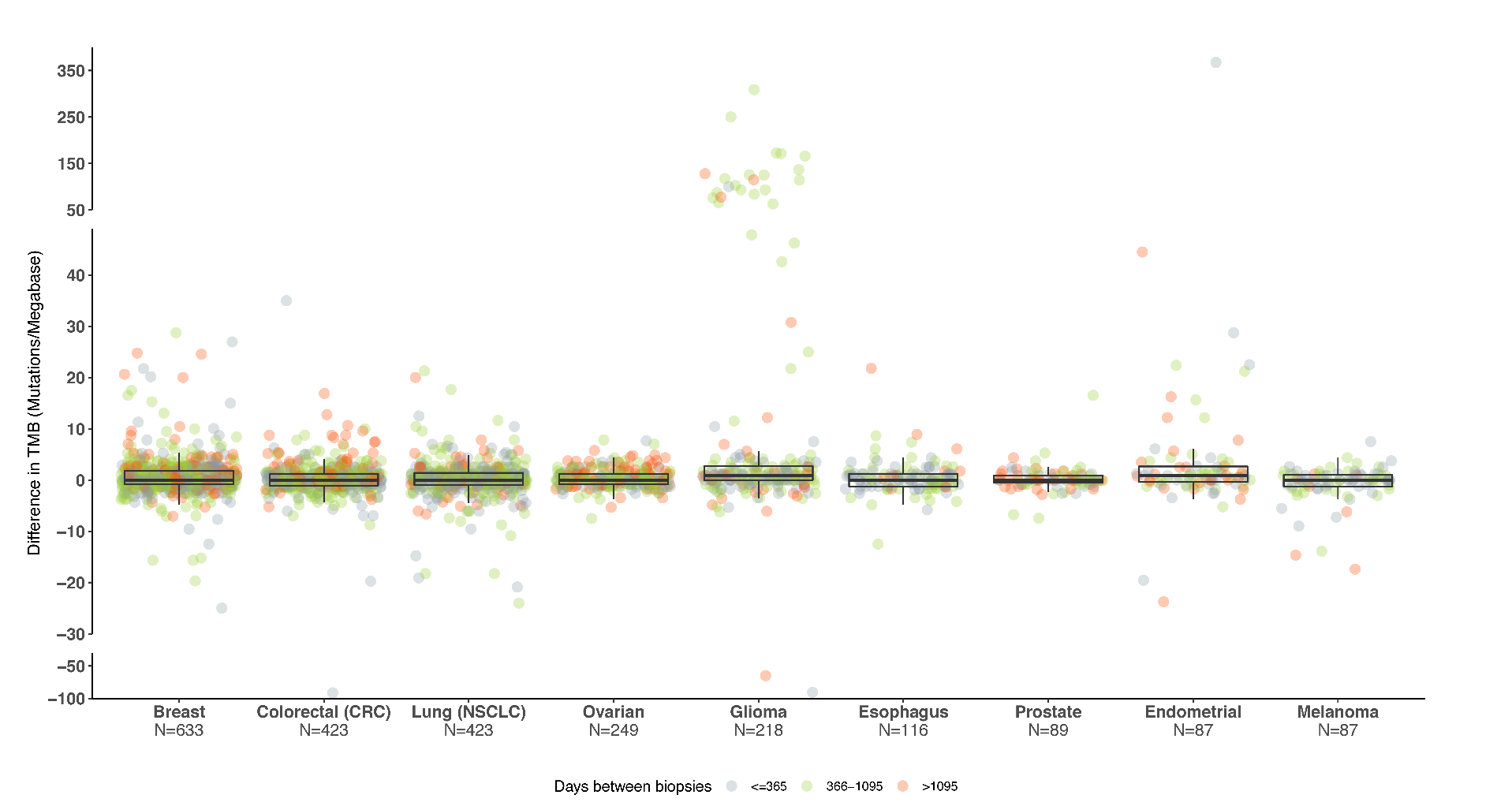


## Figure S4: Difference in TMB from paired biopsies in Foundation Medicine Cohort

The difference in TMB in paired samples from the Foundation Medicine cohort in **Figure 1**, across all elapsed time interval categories (≤ 365 days, 366 – 1095 days, > 1095 days), are shown. The fact that all the data points, regardless of time “bin”, appear to be superimposed on one another illustrates how TMB does not tend to change with time.
