# Supplementary material for: Intra-patient stability of tumor mutational burden from tissue biopsies at different time points in advanced cancers
Source: Genome Med. 2021 Oct 12;13:159. doi: 10.1186/s13073-021-00979-8 (PMC8513181; doi:10.1186/s13073-021-00979-8)
Supplement: Supplementary file 3 — Additional file 3:. PREDICT Study Protocol and Consent Form. Study Protocol. Consent Form [file 13073_2021_979_MOESM3_ESM.docx]

# **Additional File 3: PREDICT Study Protocol and Consent Form**

## **Study Protocol**

**UCSD Human Research Protections Program**

**New Biomedical Application**

**RESEARCH PLAN**

1. **PROJECT TITLE**

Moores PREDICT (Profile Related Evidence Determining Individualized Cancer Therapy)

1. **PRINCIPAL INVESTIGATOR**

Razelle Kurzrock, M.D.

Professor of Medicine

UCSD Moores Cancer Center

1. **FACILITIES**

The study will be performed at the following facilities:

- UCSD Moores Cancer Center, 3855 Health Sciences Dr., La Jolla, CA 92093
- UCSD Medical Center-Hillcrest, 200 West Arbor Dr., San Diego, CA 92103
- UCSD Medical Center-La Jolla (Jacobs Medical Center), 9300 Campus Point Dr., San Diego, CA 92037
- UCSD Medical Center-Vista, 910 Sycamore Street, Suite 102, Vista, CA 92081
- UCSD Medical Center-Encinitas, 1200 Garden View Road, Encinitas, CA 92024
- UCSD Perlman Medical Offices, 9350 Campus Point Drive, San Diego, CA 92037
- Koman Family Outpatient Pavilion, 9400 Campus Point Drive, La Jolla, CA 92037
- Rady Children’s Hospital - San Diego (RCHSD), 3020 Children’s Way, San Diego CA 92123
- Rady Children’s Hospital Institute for Genomic Medicine (RCIGM) Biorepository , 7910 Frost Street, Room 435, San Diego, CA. 92116
- Eisenhower Medical Center (EMC), 39000 Bob Hope Drive, Lucy Curci Cancer Center, Rancho Mirage, CA 92270

1. **ESTIMATED DURATION OF THE STUDY**

The estimated duration of this study is 10 years.

1. **LAY LANGUAGE SUMMARY OR SYNOPSIS**

This project will be used to study personalized cancer therapy including response and toxicity.

Personalized cancer therapy is the practice of making decisions about what kind of treatment patients should receive based on the genetic makeup of their tumor. Genes in the body code for certain characteristics such as height, eye and hair color. In tumors, there may be genes that have become abnormal and cause the cancer to grow. Researchers believe that abnormal genes may affect how individuals respond to cancer treatments. For example, a cancer patient with a specific gene abnormality may respond better to treatment than a patient without that abnormality. This is especially relevant if a drug has been developed that targets a specific gene abnormality. In that case, giving the drug to patients whose tumors have that abnormality might be more effective than giving it to patients whose tumors do not have that specific abnormality.

This study will collect information from patient medical records about the tests and treatments they have received, or will receive, for their cancer. This information will help the researchers describe if patients actually do better when they are treated according to the genetic makeup of their tumors.

This study will also perform research tests in the laboratory on tissue, blood or urine patients have provided. These laboratory tests will include creating a "profile" of their specimens, which will describe unique characteristics about the genes involved in their cancer. The tests will also help researchers look for biomarkers that may help predict how people respond to treatment. Biomarkers are proteins or chemicals in your body that may be indicators of disease status.

1. **SPECIFIC AIMS**

We will begin to explore the outcomes of patients undergoing cancer treatment when treatment either targets patient-specific tumor molecular aberrations or does not.

Aim 1: To conduct a retrospective analysis of treatment outcomes and toxicity, and pharmacodynamics and pharmacokinetics if available, in cancer patients who had a molecular profile analysis performed.

Aim 2: To prospectively examine outcomes of physician choice in treating patients who have “actionable” molecular aberrations and to characterize the molecular profiles, and pharmacodynamic and pharmacokinetic information if available, of cancer patients and their association with clinical outcomes.

Aim 3: To perform multi-modality analysis of patient specimens for molecular profiling and biomarker discovery, pharmacodynamics and pharmacokinetics.

Aim 4: To retrospectively examine the relationship between host factors such as MHC-related genes that can determine response to therapy, especially immunotherapy.

Aim 5: To analyze whether or not patients entered on clinical trials based on their molecular results or other factors, or if they screen failed or were not enrolled, as well as clinical and related factors correlating with enrollment, screen failure and failure to enroll.

1. **BACKGROUND AND SIGNIFICANCE**

The cornerstone for determining cancer treatment lies in classifying tumors by histology. A histologic diagnosis is based on the appearance of the tumor under a microscope and closely correlates with the organ in which the tumor originates. Once a pathologic diagnosis is made, drugs are chosen based on that diagnosis. For example, if cancer originates in the breast, patients are treated using drugs known to impact breast cancer; if it originates in the lung, drugs for lung cancer are chosen. With this system, response rates using single agents to treat the most common types of metastatic cancer usually remain around 0 to 30%, regardless of the drug.

It is now clear that common aberrations in cancer such as RAS, RAF, PI3K, and KIT mutations, MET amplification, and others occur in a variety of different cancers. Furthermore, the presence of these aberrations may be critical to achieving response, especially when using agents with specific molecular targets. For instance, KIT kinase inhibitors are effective in patients with KIT kinase mutations.^1,2,3^ Similarly, preclinical and emerging clinical data suggest that PI3K inhibitors may be most effective in patients with PI3K or PTEN mutations; RAF inhibitors, in patients with RAF mutations; MEK inhibitors, in patients with RAS or RAF mutations, and so forth.^4-9^ In some cases, matching patients with targeted therapies has resulted in transformative changes. For instance, treatment of chronic myelogenous leukemia, a disease driven by an aberrant *BCR-ABL* kinase, with imatinib (a *BCR-ABL* kinase inhibitor) increased median survival from four years to 20 to 25 years.^10^ *These results suggest that cancer needs to be reclassified based on its molecular features in addition to its histology, in order to optimize treatment regimens using targeted agents.*

***Previous Experience with a Personalized Medicine Approach***

The allocation of patients with cancer to treatment with specifically targeted therapies has proven to be efficacious; however, historically this approach involved molecular screening for a single aberration and matching with a single targeted drug. Because most aberrations of cancer-related genes are rare, sequential single-aberration screening is unlikely to be practical in clinical practice. Rather, profiling for multiple aberrations and assigning an appropriately targeted drug or drugs, from a portfolio of agents, was felt to be needed. This strategy was taken for patients with advanced cancer who were treated in the Clinical Center for Targeted Therapy. In 2008, Dr. Kurzrock and colleagues initiated a personalized medicine program for patients referred to the Phase I Clinic at The University of Texas MD Anderson Cancer Center (Houston, TX). ^11^ The goal was to observe whether molecular analysis of advanced cancer and use of targeted therapy to counteract the effects of specific aberrations would be associated with improved clinical outcomes. Molecular analysis was conducted in the MD Anderson Clinical Laboratory Improvement Amendments (CLIA)-certified Molecular Diagnostics Laboratory for a panel of twelve genes. Patients whose tumors had an aberration were treated with matched targeted therapy, when available. Treatment assignment was not randomized. The clinical outcomes of patients with molecular aberrations treated with matched targeted therapy were compared with those of consecutive patients who were not treated with matched targeted therapy.

Of 1,144 patients analyzed, 460 (40.2%) had 1 or more of the aberrations tested. In patients with 1 molecular aberration, matched therapy (n = 175) compared with treatment without matching (n = 116) was associated with a higher overall response rate (27% vs. 5%; P < 0.0001), longer time-to-treatment failure (TTF; median, 5.2 vs. 2.2 months; P < 0.0001), and longer survival (median, 13.4 vs. 9.0 months; P = 0.017). Matched targeted therapy was associated with longer TTF compared with their prior systemic therapy in patients with 1 mutation (5.2 vs. 3.1 months, respectively; P < 0.0001). In multivariate analysis in patients with 1 molecular aberration, matched therapy was an independent factor predicting response (P = 0.001) and TTF (P = 0.0001). Keeping in mind that the study was not randomized and patients had diverse tumor types and a median of 5 prior therapies, *these results suggest that identifying specific molecular abnormalities and choosing therapy based on these abnormalities is relevant in clinical trials.*

***Current Study Approach***

Classic oncology clinical trials examine a single or restricted number of tests, or a single or restricted number of drugs. However, experience from molecular profiling of patients suggests that each patient with metastatic disease may have a unique set of molecular rearrangements. Indeed, of the first 50 patients that have had molecular profiling done as part of their standard of care at the UCSD Moores Cancer Center, using a CLIA 180 gene panel, no two patients have had the same molecular profile. Furthermore, examining sixty patients with advanced breast cancer profiled either at UCSD or MD Anderson showed that each patient had one to eight aberrations and no two patients were identical even with the same histologic class (breast cancer). Patients have different sets of genes that are aberrant and different aberrations within those genes (e.g., amplifications versus mutations versus rearrangements, and mutation site within the same gene may differ from patient to patient as well). These observations are echoed by the literature as well. This suggests that the classic paradigm of studying a group of patients with a single or restricted therapeutic regimen or using a single or restricted set of genomic markers does not fit the reality of tumors as revealed by advancing molecular tools.

The current study will expand upon the results described at MD Anderson above. First, molecular technology has advanced considerably since 2008 in the depth of information available, turnaround time and reduced costs. For example, as of 2013 FoundationOne^TM^ provides next generation sequencing in routine cancer specimens for the entire coding sequence of 236 cancer-related genes in 21 days or less. This test can be ordered as a standard diagnostic panel and is performed in a CLIA certified manner.

The goal of the current PREDICT program is to study the molecular profiling of cancer patients and its association with physician’s choice of treatment and patient outcomes, as well as pharmacodynamic and pharmacokinetic parameters when available that may be relevant to the impact of drug targeting. This is a descriptive, exploratory, and hypothesis-generating study.

New host factors have been shown to be involved in response to drugs. For instance, high tumor mutational burden and specific tumor mutations such as those in mismatch repair genes may affect responses to immunotherapy. Checkpoint blockade immunotherapy reactivates the immune system, but the immune system must then be able to differentiate tumor from normal cells. It can do so by recognizing neo-antigens, the product of the tumor mutanome. Very recently, it has been recognized that host factors such as MHC-related regions determine how well neo-antigens are presented. These factors may affect cancer prognosis and furthermore, it is the interaction between the host and genomic alterations that can determine response to checkpoint blockade as well as, perhaps, targeted therapies and chemotherapy. Hence we will also study the impact of these genetic factors on prognosis and therapeutic outcome.

1. **PROGRESS REPORT/PRELIMINARY STUDIES**

Not applicable.

1. **RESEARCH DESIGN AND METHODS**

This is a non-therapeutic, correlative study of personalized medicine with retrospective and prospective components. Patient medical records will be examined for results of molecular profiling obtained through standard of care testing to help understand, in a descriptive fashion, how well molecular testing might predict response to therapy. Patient outcome parameters including, but not limited to, tumor response, time to treatment failure, patient survival, and toxicity will be analyzed, as well as pharmacodynamic (PD) and pharmacokinetic (PK) data when available. This study will also include research-related testing of tissue, blood, or urine specimens via a variety of simple or advanced techniques such as molecular, proteomic, and metabolic analyses for biomarker discovery or for PK and PD parameters.

*Retrospective Review*

**Aim 1** of the study is to conduct a retrospective chart review on patients seen in the UCSD Health System, EMC, and RCHSD who have a diagnosis of cancer or cancer-related condition prior to August 23, 2013. Medical records will be reviewed for documented molecular profiling that have had any kind of cancer treatment after the profiling occurred. Treating physicians will be able to provide guidance to study staff by directing them to patients who most likely have had molecular profiling performed. When available, their molecular profile will be collected along with the type and stage of cancer, prior and current cancer treatments and outcomes, general medical history, and demographics. We will determine if patients were matched to therapy or not by the physician on the basis of their molecular aberration, and the outcome. Efficacy outcome parameters will be response rates, progression-free survival, overall survival, and comparison of progression free survival on the therapy used after molecular profiling to that on previous standard therapy (hence each patient can be their own control). Similar to a previously published report of toxicity risks in oncology patients treated in phase I clinical trials^12^, safety outcome parameters will be serious toxic effects, primarily grades 3 to 5 toxicity (such as those toxic effects evaluated as being related to study drug, both in the dose-limiting toxicity window and throughout the duration of treatments), but may also include less serious chronic toxicity. Toxicity will be assessed using the National Cancer Institute Common Terminology Criteria for Adverse Events (NCI CTCAE), version 4.0.

Data extracted from the patient’s medical record will be coded with a unique study number instead of the patient’s name or identifying information. A log that links the patient’s name and identifies to the study number will be maintained in a secure database as described in Section 16. Patient baseline characteristics will be described and the molecular data will be associated with treatment outcomes (see Data Analysis and Statistical Considerations below).

*Prospective Review*

Patients seen in the UCSD Health System, EMC, or RCHSD with a diagnosis of cancer or cancer-related condition at the time of study activation or later will be asked to sign an Informed Consent document describing study Aims 2 and 3 in order to allow access to their medical records and permission in some cases to perform molecular profiling on existing or collected specimens. If a patient is < 18 years of age, consent from a legal guardian will be obtained with written assent from patients > 7 years of age when indicated.

At the time of a scheduled routine blood draw, clinical laboratory personnel or a qualified clinical research associate will obtain up to an additional 100 mL of blood from adult patients or age/weight appropriate volume for pediatric patients from consented subjects for the purpose of this specimen collection project. Subjects that have recent laboratory testing indicating hemoglobin levels less than 7 grams/dL (the current threshold for giving a patient a blood transfusion) will not be allowed to provide blood for research use. No more than 100 mL of blood will be collected for research use in an 8-week period.

Access to the medical record and molecular profiling of specimens for patients identified from the retrospective portion, but are considered lost to clinical follow-up, will also be included. Patients lost to clinical follow-up have not been seen in the UCSD Health System, EMC, or RCHSD from at least one year and do not have an appointment scheduled. Please refer to Item 12 for the waiver of informed consent for those subjects lost to clinical follow-up

**Aim 2** of the study is to prospectively examine outcomes of physician choice in treating patients who have “actionable” molecular aberrations and to characterize the molecular profiles of cancer patients and their association with clinical outcomes.

A patient’s tumor may be considered matched with a targeted therapy if a drug is known to inhibit the aberration at low nmol/L concentrations or works through other mechanisms of action including, but not limited to, suppression of gene/protein expression, epigenetic modification, or effects on proteasomal degradation. The physician choice of treatment may vary over time based on their assessment of the patient.

Patient medical records will be reviewed during the course of this study to collect molecular profiling results performed as part of their routine medical management (independent of the current study) and subsequent treatment outcomes. We will also collect the results of molecular and biomarker profiling performed as part of other IRB-approved clinical studies and treatment outcomes when the information is in the patients’ medical records. CLIA-approved molecular profiling results are scanned into the EMR and can be found in the Media section (even when performed as part of other IRB-approved clinical studies). We will select only those cases that include the results from molecular profiling. We estimate that about two hundred medical records will include scans from molecular profiling for the retrospective part of the study.

Clinical outcome data will continue to be collected on participating subjects as long as they are being seen in the UCSD Health System, EMC, or RCHSD (or by review of the Social Security Index for survival data) for the duration of this study. Data extracted from the patient’s medical record will be coded with a unique study number instead of the patient’s name or identifying information.

We will determine if patients are matched to therapy or not by the physician on the basis of their molecular aberration, and the outcome. Outcome parameters will include, but not be limited to, response rates, progression-free survival, overall survival, and toxicity. The association of molecular data with treatment outcomes will be described as listed in the Data Analysis section below.

Since molecular profiling is not currently performed on all patients, study staff will first focus efforts on those patients likely to have had profiling performed or those patients whose treating physicians contact the study staff about potential eligibility. For example, a biweekly molecular tumor board was initiated at the Moores Cancer Center in December 2012 to cover topics in molecular profiling and personalized medicine. These meetings will provide an opportunity to identify candidate patients and bring an awareness of the PREDICT program for Cancer Center physicians to discuss with the study staff. Study staff will access patient medical records at a minimum of once per year in order to identify those patients having received molecular profiling. For those patients with profiling performed, either as part of routine cancer care or performed as part of this study, access of their medical records may occur more frequently (e.g., quarterly) to follow treatment outcomes.

**Aim 3** of the study is to perform multi-modality analysis of patient specimens for molecular profiling and biomarker discovery. This testing will be *optional* and patients will be informed of this option in the Informed Consent document (unless lost to clinical follow-up). Patient-derived samples, from patients being followed by UCSD, EMC, or RCHSD, utilized for research testing will be obtained from 1) clinical specimens archived by UCSD Health System Pathology or from specimens collected via the HRPP #181755 (previously #090401) Biorepository protocol “Collection and Banking of Biological Samples for Use in Biomedical Research”, 2) discarded specimens, or 3) from specimens collected for this protocol (optional in consent) or 4) clinical specimens archived by the RCHSD Biorepository (UCSD HRPP No. 121644). Examples of discarded samples would be urine, stool, tracheal aspirate, cerebral spinal fluid (CSF), or the fluid removed routinely from patients with ascites or pleural effusions. For subjects consenting to the optional urine collection, urine collection cups may be sent home with the participant for return to the clinic in person or via a shipping courier such as FedEx. If patient is having bronchoalveolar lavage (BAL) as part of routine care, extra BAL fluid can be sent off for genomic analysis. Specimens collected for the Biorepository protocol require separate consent, which is part of that protocol. Storage and use of samples will comply with protocol #181755 (previously #090401). Specimens used for analysis will be labeled with the patient’s de-identified study number and collection date.

As a crucial part of the study is correlating findings in specimens with outcome data, researchers in this protocol will be able to identify patients and collect data from their electronic medical records.  All data will be stored and protected in a HIPAA compliant manner.

Analysis of specimens from the patients lost to clinical follow-up will not be optional for the patients, but rather will be at the researcher’s discretion. Biological samples collected from patients lost to clinical follow-up are not participants of the Biorepository and their tissue samples were not collected as part of another research project.

The specimens, DNA, and their derivatives may have significant therapeutic or commercial value. The Informed Consent form contains this information and informs the subject that there is the potential for financial gain by UCSD, the investigator or a collaborating researcher or entity.

Specimens may also be analyzed for infectious organisms as these organisms may affect cancer outcome as well as response to immunotherapeutics.

Patient-derived samples may be used and data analyzed for a variety of purposes including the following:

1. Tumor markers such as CA125, CEA, CA15.3, CA19.9, αFP, PSA, inhibins, calcitonin, thyroglobulin, chromagranin and others.
2. Circulating tumor cells (CTC) and analysis of CTC at the DNA, RNA, and protein levels.
3. Serum antigens using three different assays depending on antibodies to be used: ELISA, Luminex, and Reverse phase protein array (RPPA).
4. Circulating or tumor DNA.
5. Next generation or other DNA sequencing.
6. Transciptome analysis.
7. Metabolome analysis.
8. Proteomic analysis.
9. Spliceosome analysis.
10. Pharmacodynamic analysis.
11. Pharmacokinetic analysis.
12. Other cancer assays.
13. Assays for infectious organisms of sputum, buccal, nasal, nasopharyngeal, fecal, and other non-invasive swabs.
14. Assays for infectious of organisms of bronchoalveolar lavage or other fluid samples collected during procedure already being performed for another indication.
15. Analysis of infection-related assays performed as standard of care.

To take into consideration rapidly evolving technologies, analysis of patient samples will be performed in a variety of locations depending on the appropriate expertise needed. Availability of potential funding or collaborations will factor into which tests will be performed and the patient’s treating physician can provide input to the study investigators as to which types of tests might be suitable or most informative for their patients. Due to the costs of the assays and technical issues of specimen availability, not all assays can or will be performed on all subjects.

The results of research-related molecular profiling and biomarker testing will be compared to the patient’s contemporaneous treatment regimen extracted from their medical records to see if their therapy is by chance matched (or unmatched). These treatment outcomes will also be collected for analysis.

**Aim 4** is to retrospectively examine the relationship between host factors such as MHC-related genes that can determine response to therapy, especially immunotherapy, in subject’s data available prior to November 15, 2018. Data elements described in Aim 1 will also be collected retrospectively for patients in Aim 4.

Aim 4 is added as we have learned more about the role of genomics and genetics in prognosis and determining therapy response and it is a natural extension of the other three aims.

Host factors have been shown to be involved in response to drugs in addition to genomic factors. For instance, high tumor mutational burden and specific tumor mutations such as those in mismatch repair genes may affect responses to immunotherapy. Checkpoint blockade immunotherapy reactivates the immune system, but the immune system must then be able to differentiate tumor from normal cells. It can do so by recognizing neo-antigens, the product of the tumor mutanome. Very recently, it has been recognized that host factors such as MHC-related regions determine how well neo-antigens are presented. These factors may affect cancer prognosis and furthermore, it is the interaction between the host and genomic alterations that can determine response to checkpoint blockade as well as, perhaps, targeted therapies and chemotherapy. Hence we will also study the impact of these genetic factors on prognosis and therapeutic outcome. It should be noted that MHC data is available from routine testing or from sequencing already performed. We will retrospectively analyze the results on up to 5,000 UCSD patients and up to 200 RCHSD patients with available data and determine how the interaction between the host factors such as MHC-related genes and genomics affects prognosis and therapeutic outcome.

**Aim 5** of the study is to analyze whether or not patients entered on clinical trials based on their molecular results or other factors, or if they screen failed or were not enrolled, as well as clinical and related factors correlating with enrollment, screen failure and failure to enroll. One of the most important issues encountered in patients is whether they can enroll on clinical trials. This is especially relevant to patients who have had molecular testing. Understanding these issues is a natural extension of the other aims.

Information will be collected from patients already enrolled in PREDICT who screen failed another study after inclusion in PREDICT (PREDICT does not rule out enrollment in other studies). This demographic data is collected from the electronic medical record and does not differ from collection of other demographic data under PREDICT.

This data can be collected under the other aims, but is clarified here under Aim 5. Data will include any reasons that patients failed to be treated ok as study, e.g. their refusal, health deterioration, did not meet eligibility, investigational drug study discontinued, etc. Any information collected will be de identified.

Information from patients who decline enrollment in PREDICT will not be kept, beyond identifiers kept so that they are not re-approached after having declined any participation.

Return of Results

When non-CLIA-certified assays are performed, results will not be entered in the patient’s medical record and results may be returned to study participants only with the understanding that the results were gained from investigative procedures. It is possible that incidental germline DNA findings with health-related consequences for the subject and/or their family may be found; however, these findings may not be returned to the patient or their treating physician.

Incidental findings from this sequencing, or any other research sequencing, will be handled following the latest ACMG guidelines (current reference Genetic Medicine 2013, Jul; 15(7):565-74; PMID 23788249) in coordination with our Family Cancer Genetics. No additional clinical testing will be performed without documented clinical consent.

When CLIA-certified testing presents germline DNA findings, results may be returned to the patient’s treating providers (oncologist and/or genetic counselor) if the patient has indicated in the Informed Consent document that they would like their treating providers notified of possible “inherited risks of getting cancer”.

**Data Analysis and Statistical Considerations**

Descriptive statistics will be used. No formal statistical hypothesis testing will be conducted. An exploratory analysis will be performed.

We are anticipating enrollment of up to 10,000 patients.

For characterizing the molecular profile, descriptive statistics and exploratory data analysis will be performed first. Categorical data will be described using contingency tables. Continuously scaled measures will be summarized with descriptive statistical measures (i.e., mean (± s.d.) and median (range)). Distribution plots such as histograms and box plots will be applied. For correlating marker profiles with response to treatment, univariate and multivariate (multiple covariates) logistic regression will be used, with the response status taken as the outcome variable. Multiple covariates include disease type, histology, stage, treatments, doses, and patients’ medical demographical variables will also be considered in modeling the outcome variable. Chi-square test or Fisher’s exact test (when sample size is small) will be applied to test the association between two categorical variables. Pearson’s or Spearman’s correlation coefficients will be calculated whenever appropriate to quantify the association between two continuous variables. We will estimate the distributions of time-to-event outcomes using Kaplan-Meier curves and compare these distributions among groups using the log rank test. As appropriate, we will compare progression-free survival on treatments that patients are given based on molecular tests to the progression-free survival seen in those patients on prior therapy, or to progression-free survival of patients whose treatment was not based on molecular testing. Similar assessments will be performed for time-to-treatment failure, response and survival as appropriate. Cox proportional hazards models will be used to test for covariate effects on survival endpoints in the presence of multiple covariates. Due to the fact that serial samples will be taken over time, proper methods for analyzing longitudinal data such as the repeated measures ANOVA, mixed models, and the generalized estimating equations methods will be used to address the correlation among the measurements. In addition, marker measures over time can be treated as time varying covariates in the survival analysis for analyzing time to event outcomes. Finally, we will analyze, descriptively, observed correlations between specific molecular aberrations and response and toxicity.

We are aware of multiple statistical tests being performed. However, no multiple comparison adjustments are proposed because of the exploratory nature of the PREDICT Program. The strength of the finding will be gauged by the confidence interval estimation and results will need to be confirmed by future studies. If a biomarker is to undergo future investigation, its p value threshold of interest will be < 0.001.

Sample Collection from RCHSD

Once informed consent has been obtained, samples are collected and transported to RCHSD-UCSD Biorepository for processing, de-identification and storage. Each participant will be assigned a unique study ID and each sample will be labeled with participant ID and sample number. Samples will be logged into Velos eSample system. Clinical details will be obtained by Dr. Kuo from the EMR and will not be held at the RCIGM Biorepository. The RCIGM Biorepositorywill maintain only basic demographic information e.g. DOB, age, sex, race, ethnicity.

Sample Collection from EMC

Once informed consent has been obtained, samples are collected and transported to UCSD Biorepository for processing, de-identification and storage. EMC does not have biorepository on site and will be utilizing the UCSD Biorepository as necessary. Each participant will be assigned a unique study ID and each sample will be labeled with participant ID and sample number. Samples will be logged into Velos eSample system. Clinical details will be obtained by Dr. Tsai from the EMR and will not be held in the biorepository. The biorepository will maintain only basic demographic information e.g. DOB, age, sex, race, ethnicity.

1. **HUMAN SUBJECTS**

We approximate that an average of ten patient charts may be reviewed each week up to four years, therefore, expected accrual is up to 10,000 patients.

Eligibility

All patients with a diagnosis of cancer or cancer-related referred to a UCSD Health System facility, EMC, and RCHSD are eligible. Patients or their legal guardians must be willing and able to provide written informed consent to participate in the prospective part of the study unless the patient has been lost to clinical follow-up.

Withdrawal of Patients

A patient is free to withdraw from the study at any time for any reason without prejudice to his/her future medical care by the physician or at the institution. If the patient later decides that he/she does not want their future clinical data abstracted into the study database, he/she will tell this to Dr. Kurzrock or study delegate who will instruct all study staff to stop any additional data abstraction for this patient. The patient may also request that any data already abstracted into the study database be removed. The informed consent document explains that unused data will be removed from the research database unless it has already been analyzed for the study Aims.

If the patient later decides that he/she does not want their specimens collected to be used for this study, he/she may tell this to Dr. Kurzrock or study delegate who will use her/his best efforts to stop any additional specimen analysis and/or to destroy the specimens if it is so requested. The informed consent document explains that, in some cases, it may be impossible to locate and stop such future research once the specimens have been sent out for analysis.

1. **RECRUITMENT**

For the retrospective chart review component of the study:

Patients will be identified from those having a diagnosis of cancer or cancer-related condition in their medical record seen at a UCSD Health System facility, EMC, or RCHSD prior to the release date of this study’s initial IRB approval or prior to November 15, 2018 under Aim 4.

A request to waive the requirement to obtain informed consent is being requested for the retrospective chart review data collection and analysis. The investigator believes this part of the study meets the following requirements for this request per 46 CFR 46.116:

1. The research involves no more than minimal risk to the participants.
2. The waiver or alteration will not adversely affect the rights and welfare of the participants.
3. The research could not practicably be carried out without the waiver or alteration. These patients have already been treated.
4. Whenever appropriate, the participants will be provided with additional pertinent information after participation.

A waiver of HIPAA Authorization is being requested for this retrospective portion of the study. The investigator believes this study meets the following requirements for this request per 46 CFR 164. 512(i)(2)(ii):

1. The use or disclosure of protected health information (PHI) involves no more than minimal risk to the privacy of individuals;
2. The project could not practicably be conducted without a waiver; and
3. The project could not practicably be conducted without use of PHI.

Further, the privacy risks are reasonable relative to the anticipated benefits of research, as the importance of the knowledge that may reasonably be expected to result outweighs the minimal risk posed to subjects. Section 14, Risk Management Procedures, of the Research Plan includes an adequate plan to protect identifiers from improper use and disclosure and justification for retaining identifiers. PHI will not be re-used or disclosed for other purposes and, whenever appropriate, the subjects will be provided with additional pertinent information after participation. Identifiers will be destroyed by the PI at the completion of the research. Only de-identified data will be kept.

For the prospective component of the study:

Patients will be recruited from those seen in the UCSD Health System, EMC, and RCHSD with a diagnosis of cancer or cancer related condition.

For the purposes of pre-screening ONLY: a waiver of Consent is being requested as the investigator believes the pre-screening to be used for recruitment in this study meets the following requirements for this request per 45 CFR 46.116:

1. The (pre-screening) research involves no more than minimal risk to the subjects;
2. The waiver or alteration will not adversely affect the rights and welfare of the subjects;
3. The (pre-screening) research could not practicably be carried out without the waiver or alteration; and
4. Whenever appropriate, the subjects will be provided with additional pertinent information after participation.

A *partial* waiver of HIPAA authorization is also being requested for access to PHI for purposes of pre-screening ONLY since Protected Health Information (PHI) will be accessed via the hospital’s medical record database and scheduling system (e.g., CPRS) *prior* to contacting the potential subject about the research study when designated study personnel review subject records in order to identify potentially eligible subjects.

No written record of the pre-screening information will be created. There will be no direct contact of the potential research subject by the pre-screener (i.e., study staff). The pre-screener will then ask the subject’s treating physician to approach the subject. The treating physician will further discuss the research study with the potential subject and ask whether they would like to be contacted by study staff to discuss the trial (i.e., counseling) and/or provide the potential subject with the study staff’s contact information. Eligibility may be formally determined at the time of counseling, but any research-specific screening procedures will only be performed after informed consent is obtained and a standard, stand-alone HIPAA authorization form is signed.

Standard HIPAA authorization to collect research data from the subject's medical record will be obtained at the time of informed consent.

The treating physician or members of the treating team will discuss the research study with the potential patient and ask whether they would like to be contacted by study staff to discuss the study and/or provide the potential patient with the study staff’s contact information. All research-specific procedures will only be performed after informed consent is obtained and a standard, stand-alone HIPAA authorization form is signed.

Clinical Trials Navigator

The Clinical Trials Office will employ a Clinical Trials Navigator (CTN) to assist with the navigation and/or recruitment of patients to clinical trials. The CTN will have a working knowledge of the protocol eligibility requirements and enrollment opportunities for trials available through the CTO.

The CTN will access patient medical records facilitating the above detailed pre-screening process, act as contact for subject referrals from patients or investigators, and will be listed as the clinicaltrials.gov primary contact for investigator-initiated trials.

Other Recruitment Methods

Recruitment methods may include presenting the basic trial information as detailed below in a written format (e-mail, flyer, or letter) to the research and/or clinical staff of MCC, presentation at Disease Team Meetings, presentation at MCC Tumor Boards, and postings in e-mailed newsletters such as The MCC Reporter.

1. The title; purpose of the study;
2. Protocol summary;
3. Basic eligibility criteria;
4. Study site location(s); and
5. How to contact the site for further information.

Eisenhower has requested the use of a patient brochure and summarized clinicaltrials.gov listing to place through-out the cancer center, in clinic exam rooms and in the patient resource center.

1. **INFORMED CONSENT**

For the retrospective component of the study:

A request to waive the requirement to obtain informed consent is being requested for the retrospective chart review data collection and analysis. The investigator believes this part of the study meets the following requirements for this request per 46 CFR 46.116:

1. The research involves no more than minimal risk to the participants.
2. The waiver or alteration will not adversely affect the rights and welfare of the participants.
3. The research could not practicably be carried out without the waiver or alteration. These patients have already been treated.
4. Whenever appropriate, the participants will be provided with additional pertinent information after participation.

A waiver of HIPAA Authorization is being requested for this retrospective portion of the study. The investigator believes this study meets the following requirements for this request per 46 CFR 164. 512(i)(2)(ii):

1. The use or disclosure of protected health information (PHI) involves no more than minimal risk to the privacy of individuals;
2. The project could not practicably be conducted without a waiver; and
3. The project could not practicably be conducted without use of PHI.

Further, the privacy risks are reasonable relative to the anticipated benefits of research, as the importance of the knowledge that may reasonably be expected to result outweighs the minimal risk posed to subjects. Section 14, Risk Management Procedures, of the Research Plan includes an adequate plan to protect identifiers from improper use and disclosure and justification for retaining identifiers. PHI will not be re-used or disclosed for other purposes and, whenever appropriate, the subjects will be provided with additional pertinent information after participation. Identifiers will be destroyed by the PI at the completion of the research. Only de-identified data will be kept.

For the prospective component of the study:

Patients or their legal guardians will be asked in a private location to provide written consent using IRB-approved forms prior to performing any study related procedures. The investigator or study coordinator will describe the study, including detailed information about risks and benefits, to potential patients. The investigator or study coordinator will provide potential patients with an IRB-approved consent. Patients will be given ample time to read the consent form at the same visit or may take it with them to read at another time. Potential study patients will be given the opportunity to ask and receive answers to all questions they may have about the study, its risks and benefits, or the consent form itself before signing the consent form. As this research is subject to HIPAA privacy rule provisions, participants will also be requested to sign a separate authorization for the use of protected health information (i.e., HIPAA form specific to the research study). The investigator or study coordinator will obtain informed consent in a language understood by the prospective participant or their legally authorized representative, using certified translations of study documents and qualified translators, where applicable.

Patients who fulfill the eligibility criteria will be offered further participation in this study. Only patients who have been consented and provided HIPAA authorization (unless lost to clinical follow-up) will have identifiers or linked information (e.g., patients initials, study numbers, etc.) recorded on the Screening/Enrollment Log.

A copy of the signed informed consent and HIPAA authorization will be placed in the research patient’s medical record. The original consent/assent/permission form and HIPAA authorization will be retained in the master research file.

Copies of the HIPAA authorization and Informed consent that will be used on this study are attached to this IRB application for review.

Prospective Subjects Lost to Clinical Follow-up

A request to waive the requirement to obtain informed consent is being made for the data collection, sample collection and testing, and data analysis of subjects lost to clinical follow-up. Subjects who have not been seen in UCSD’s Health System, EMC’s facility, or RCHSD’s clinic for at least one year and do not have a follow-up appointment scheduled will be considered lost to follow-up. Subject’s samples collected under this waiver would have been collected during the course of the subject’s routine care and not a part of research including the participation in the UCSD Biorepository. The investigator believes this portion of the study meets the following requirements for this request per 46 CFR 46.116:

1. The research involves no more than minimal risk to the participants;
2. The waiver or alteration will not adversely affect the rights and welfare of the participants;
3. The research could not practicably be carried out without the waiver or alteration; and whenever appropriate, the participants will be provided with additional pertinent information after participation.

A waiver of HIPAA Authorization is being requested for subjects lost to clinical follow-up. As described above, the use or disclosure of PHI involves no more than minimal risk; granting of the waiver will not adversely affect privacy rights and welfare of the individuals whose records will be used; the project could not practicably be conducted without a waiver; the project could not practicably be conducted without use of PHI. Further, the privacy risks are reasonable relative to the anticipated benefits of research, as the importance of the knowledge that may reasonably be expected to result outweighs the minimal risk posed to subjects. Section 14, Risk Management Procedures, of the Research Plan includes an adequate plan to protect identifiers from improper use and disclosure and justification for retaining identifiers. PHI will not be re-used or disclosed for other purposes and, whenever appropriate, the subjects will be provided with additional pertinent information after participation. Identifiers will be destroyed by the PI at the completion of the research. Only de-identified data will be kept.

1. The research involves no more than minimal risk to the participants.
2. The waiver or alteration will not adversely affect the rights and welfare of the participants.
3. The research could not practicably be carried out without the waiver or alteration. These patients have already been treated.
4. The project could not practicably be conducted without the use of PHI.
5. An adequate plan to protect identifiers from improper use and disclosure is included in the research proposal.
6. An adequate plan to destroy the identifiers at the earliest opportunity, or justification for retaining identifiers, is included in the research proposal.
7. The project plan includes written assurances that PHI will not be reused or disclosed for other purposes.
8. Whenever appropriate, the participants will be provided with additional pertinent information after participation.
9. **ALTERNATIVES TO STUDY PARTICIPATION**

As there is no promise of direct benefit to the participant, the alternative is not to participate.

1. **POTENTIAL RISKS**

Risks of Blood Drawing: Risks associated with drawing blood are considered minimal, but some risks include: pain, excessive bleeding, fainting or feeling lightheaded, bruising, infection (a slight risk any time the skin is broken), and multiple punctures to locate veins.

***DNA Testing:*** Release of DNA test results may present a psychological risk if it leads to the discovery of inherited genetic modification (germline mutation) with potential health consequences to the patient and their family. The Genetic Information Nondiscrimination Act prohibits health insurance carriers from denying coverage because an individual took a genetic test, or from denying coverage based on test results, and prohibits employers from using this information as the basis for employment decisions. However, at this time, there are no special protections against the use of genetic information to inform the provision of life insurance, disability insurance, or long-term care insurance.

The secure database containing medically relevant information and the database log containing subject identifiers will only be accessible by UCSD employees who are charged with protecting the integrity of the database in compliance with HIPAA laws. Participation in this study may not considered genetic testing and the results of any DNA testing performed on the participant samples will never be directly provided to the patient. However, it is possible that studies of patient’s samples may reveal an important genetic finding that could impact his/her healthcare. Such findings are considered incidental, as they are not part of the planned research and such incidental findings are not to be considered clinically valid. In some cases incidental findings may inform the patient or primary care doctor, or oncologist about a medical condition that may be treatable. In the rare circumstance where a result is found that meets the ACMG’s rigorous standards for return of incidental results (current reference Genetic Medicine 2013, Jul; 15(7):565-74; PMID 23788249), the subject will be re-identified by the study PI. If the incidental result is already known (based on chart review), no further action will be taken. If the potential result is not known, then the study PI will work with the treating physician and the Family Cancer Genetics program to facilitate referral for consideration of clinical testing. The physician and/or genetics counselor will only reveal the existence of a potentially useful research result if necessary. If the subject declines referral to genetic counseling, no further action will be taken. The exact research result will not be shared with the subject nor their treating physician. If the subject declines clinical testing no further action will be taken. If consent for clinical testing is given, the exact research result may be shared with the CLIA certified laboratory that will be conducting confirmatory testing. During genetic counseling, the patient will be informed of the risk that third party payers may deny payment for any additional clinical testing.

***Loss of Confidentiality:*** For research involving genetic or protein markers of inherited susceptibility to cancer or other disease, loss of confidentiality for this information may present a risk to the patient's or their family members' ability to obtain or retain insurance or employment, or to adopt children. There may also be risks of social stigmatization. There is a potential risk of loss of confidentiality which will be minimized as outlined in the Privacy and Confidentiality section.

1. **RISK MANAGEMENT PROCEDURES AND ADEQUACY OF RESOURCES**

The risks of loss of confidentiality will be minimized as described in the Section 16 Privacy and Confidentiality below. The PI will promptly submit reports of unanticipated problems involving risk to patients or others (UPRs) to the UCSD IRB.

1. **PRIVACY AND CONFIDENTIALITY CONSIDERATIONS INCLUDING DATA ACCESS AND MANAGEMENT**

Confidentiality of a patient’s protected health information will be maintained by the following methods: study-specific records containing protected health information, and copies of study-related medical records, will be kept in locked filing cabinets at the Moores UCSD Cancer Center, EMC, and RCHSD research administrative offices. Data extracted from the patient’s medical record and patient samples used for profile testing will be coded with a unique study number rather than the patient’s name or other identifying information.

Computers containing access to protected health information and the log that links the patient’s protected health information to their unique study number will have password-access; and screen-savers will be utilized to prevent unauthorized viewers from inadvertently seeing information.  Protected health information will be stored on secure servers. The servers supporting these studies are located inside a locked rack within the San Diego Supercomputer data center.  The facility features restricted access by means of personal codes (PINs) and biometrics, and is monitored 24 hours a day 7 days a week.   The electrical power is backed up by battery-powered uninterruptible power supplies, as well as by on-site generators.  Both servers are behind firewalls configured to allow access only to credentialed personnel within the UCSD, EMC, and RCHSD networks.  The servers run only those services necessary for current operations.  Services not needed for current operations have been disabled.  The clinical trials management system VELOS maintain an additional internal access control mechanism via user names and passwords.  Data transactions executed over our intranet are encrypted via Secure Sockets Layer.

Access to a patient’s protected health information and coded study numbers will be limited to those study personnel who need to use it to accomplish the purpose of the research, and the minimum necessary information to accomplish the purpose of the research will be collected, stored, used, and reported.  When protected health information is sent outside the University of California, San Diego, EMC, and RCHSD, it will be disclosed only to those parties listed in the patient’s authorization, and an audit trail log will be maintained of what information was sent and to whom it was sent.

Information from analyses of subject samples and the subset of the subject’s medical information will be put into databases along with information from the other research participants. These databases may be accessible by institutions and companies that are studying various diseases. Please note that traditionally-used identifying information about the subjects, such as name, address, telephone number, or social security number, will NOT be put into the database(s).

1. **POTENTIAL BENEFITS**

This trial may benefit future patients by advancing knowledge of molecular profiles and how they relate to cancer treatment. There may be no direct benefit for patients participating in this trial.

1. **RISK/BENEFIT RATIO**

The potential benefit is judged to outweigh risk; therefore, the risk/benefit ratio is in favor of benefit.

1. **EXPENSE TO PATIENT**

All procedures not considered standard of care or those that don’t fall under CLIA certification will be paid for by Moores Cancer Center or by specific grants. For instance, if exomic next gen sequencing is performed in order to better understand a patient’s disease, the lab performing that procedure may use grants or other funding to cover the costs. Standard of care tests, treatment, and patient specimen procurement is not performed as part of this protocol (see above) and hence will not result in any patient expense.

1. **COMPENSATION FOR PARTICIPATION**

There will be no payment to the patient for their participation in this study.

1. **PRIVILEGES/CERTIFICATIONS/LICENSES AND RESEARCH TEAM RESPONSIBILITIES**

Dr. Kurzrock, the principal investigator of this study, will be responsible for the overall conduct of the study, determining patient eligibility, and data analysis.

Please refer to the HRPP Facesheet and Facesheet Addendum for a list of co-investigators on this study.

Physicians listed on the HRPP facesheet are licensed physicians in the State of California with privileges at the University of California, San Diego Medical Centers. Their roles in the study will include recruitment of patients, determining patient eligibility, and data analysis. Dr. Capparelli, Dr. Schwaederle and Mr. Parish will assist with data collection and analyses.

Dr. Madlensky, the Director of the Family Cancer Genetics Program at the Moores UCSD Cancer Center, is certified by the American Board of Genetic Counseling and Licensed by the State of California. She will be responsible for returning results of inherited risk variants results from CLIA-certified genetic testing.

Study coordinators at the Moores UCSD Cancer Center will be responsible for the recruitment and consenting of patients, completing enrollment logs, maintaining source documents, and serving as the liaison with the study team.

Co-investigators at the RCHSD and the pediatric oncology faculty will be responsible for the recruitment and consenting of patients, completing enrollment logs, maintaining source documents, and serving as the liaison with the UCSD study team. RCHSD will also employee research coordinators to assist with consenting patients, specimen handling, and data collection. All participating RCHSD staff are listed in the HRPP Facesheet and Facesheet Addendum.

RCIGM Biorepository staff are responsible for collection, processing, de-identification, sample numbering and storage of biosamples obtained at RCHSD. RCIGM Biorepository staff:

Kathryn Bouic, SRA II

Linda Luo, SRA II

Co-investigators at EMC (including Henry Tsai, MD) will be responsible for the recruitment and consenting of patients, completing enrollment logs, maintaining source documents, and serving as the liaison with the UCSD study team.

The Moores UCSD Cancer Center Clinical Trials Office regulatory associate will be responsible for completing the IRB and ancillary applications, corresponding with the various committees, trial master file management and serving as the liaison with the study sites regarding study documents and IRB reporting.

Dr. Kurzrock and all the co-investigators on this study have completed the appropriate CITI training and their certificates are on file on the UCSD Cancer Center Clinical Trials Office shared network drive.

1. **BIBLIOGRAPHY**
2. Hirota et al. Gain-of-function mutations for kit in human GIST tumors. Science 279:577, 1998.
3. Demetri et al. Efficacy and safety of imatinib in advanced GIST tumors. NEJM 47:472, 2002.
4. Hodi F, Friedlander, P. Major response to imatinib in KIT-mutated melanoma. J Clin Oncology 26:2046, 2008.
5. Janku, F., J. J. Wheler, et al. (2013). "PIK3CA Mutation H1047R Is Associated with Response to PI3K/AKT/mTOR Signaling Pathway Inhibitors in Early-Phase Clinical Trials." Cancer Res **73**(1): 276-284.
6. Janku, F., J. J. Lee, et al. (2011). "PIK3CA mutations frequently coexist with RAS and BRAF mutations in patients with advanced cancers." PLoS One **6**(7): e22769.
7. Janku, F., A. M. Tsimberidou, et al. (2011). "PIK3CA mutations in patients with advanced cancers treated with PI3K/AKT/mTOR axis inhibitors." Mol Cancer Ther **10**(3): 558-565.
8. Solit DB, Garraway LA, Pratilas CA, et al. BRAF mutation predicts sensitivity to MEK inhibition. Nature 2006; 439(7074): 274-5.
9. El-Osta, H., G. Falchook, et al. (2011). "BRAF mutations in advanced cancers: clinical characteristics and outcomes." PLoS One **6**(10): e25806.
10. Chapman PB, Hauschild A, Robert C, Haanen JB, Ascierto P, Larkin J, et al. Improved survival with vemurafenib in melanoma with BRAF V600E mutation. N Engl J Med 2011;364:2507–16.
11. Westin, J. R. and R. Kurzrock (2012). "It's about time: lessons for solid tumors from chronic myelogenous leukemia therapy." Mol Cancer Ther **11**(12): 2549-2555.
12. Tsimberidou, A. M., N. G. Iskander, et al. (2012). "Personalized medicine in a phase I clinical trials program: the MD Anderson Cancer Center initiative." Clin Cancer Res **18**(22): 6373-6383.
13. Wheler JJ, Tsimberidou AM, Hong DS, Naing A, Falchook GS, Fu S, Moulder S, Stephen B, Wen S, Kurzrock R. Risk of serious toxicity in 1181 patients treated in phase I clinical trials of predominantly targeted anticancer drugs: the M. D. Anderson Cancer Center experience. Ann Oncol. 2012 Aug;23(8):1963-7.
14. **FUNDING SUPPORT FOR THIS STUDY**

This is an investigator-initiated protocol funded by UCSD Moores Cancer Center.

1. **BIOLOGICAL MATERIALS TRANSFER AGREEMENT**

Potential research collaborators outside of UCSD who approach the Cancer Center for clinical specimen will be required to complete an agreement (Material Transfer Agreement) stating that the specimens will only be released for use in disclosed research, and any specimen left over from research will either be returned to the Cancer Center or destroyed.

Unless otherwise agreed by UCSD and the collaborator, any data obtained from the use of clinical specimen will be the property of UCSD for publication and licensing, and any licensing agreement will be strictly adhered to. These outside collaborators may include for-profit biotechnology corporations interested in collaborating with UCSD investigators in research diagnostic, prognostic assay and drug development.

1. **INVESTIGATIONAL DRUG FACT SHEET AND IND/IDE HOLDER**

Not applicable.

1. **IMPACT ON STAFF**

There is a study coordinator available for this study. There will be no excess nursing burden.

1. **CONFLICT OF INTEREST (COI)**

The appropriate forms (i.e. 700U) have been submitted to the Conflict of Interest Office, via the Office of Contracts and Grants Administration (OCGA)*.*

Dr. Kurzrock is an unpaid consultant for Sequenom. A Management of Conflict Plan had been agreed to by Dr. Kurzrock and Independent Review Committee (IRC). Updated forms have been submitted to OCGA reflecting Dr Kurzrock’s unpaid consultant status.

Dr. Kurzrock has purchased equity interest as a Convertible Security in IDbyDNA. Dr. Kurzrock’s master protocol has a sub-project under an MTA with Dr. Goodman as the PI. Dr Kurzrock is the co-investigator on the MTA, she does not see or consent patients. A Management of Conflict Plan had been agreed to by Dr. Kurzrock and Independent Review Committee (IRC).

1. **SUPPLEMENTAL INSTRUCTIONS FOR CANCER-RELATED STUDIES**

This study has been approved by the Moores UCSD Cancer Center Protocol Review and Monitoring Committee (PRMC) for review.

1. **OTHER APPROVAL/REGULATED MATERIALS**

Not applicable.

1. **PROCEDURES FOR SURROGATE CONSENT AND/OR DECISIONAL CAPACITY**

**ASSESSMENT**

Not applicable.

## **Consent Form**

University of California, San Diego

Consent to Act as a Research Subject

**Moores PREDICT (Profile Related Evidence Determining Individualized Cancer Therapy)**

Dr. Kurzrock and her colleagues are conducting a research study to learn more about selecting treatment options for patients. You are being asked to take part because you are a patient with cancer or with a cancer-related condition seen in the UCSD Health System.

Your participation in this research study is voluntary. The purpose of this Informed Consent Form is to inform you about the nature of this research study so that you may make an informed decision as to whether you would like to participate. If you have any questions, please ask your study doctor or coordinator to explain any words or information that you do not understand.

**PURPOSE**

The purpose of this study is to learn more about personalized cancer therapy including response to treatment and side effects. Personalized cancer therapy is the practice of making decisions about what kind of treatment you should receive based on the genetic makeup of your tumor. Genes in your body code for certain characteristics such as height, eye and hair color. Researchers believe that abnormal genes in tumors may affect how individuals respond to cancer treatments. For example, a cancer patient with a specific gene abnormality in their tumor may respond better to a treatment that targets that abnormality than a patient without that abnormality.

This study will collect information from your medical record about the tests and treatments you have received, or will receive, for your cancer. This information will help the researchers describe whether or not patients respond better when their physicians choose to treat them according to the genetic makeup of their tumor.

This study may also perform research tests in the laboratory on tissue, sputum, body cavity fluid, blood or urine you have provided, discarded biological samples taken during your routine care that would normally be disposed of and not saved, or on blood samples or swabs collected for this study (optional).

These laboratory tests will include creating a "profile" of your specimens, which will describe unique characteristics about the genes involved in your cancer. The tests will also help researchers look for biomarkers that may help predict how people respond to treatment. Biomarkers are proteins or chemicals in your body that may be indicators of your disease status.

Participation in this study is entirely voluntary. Approximately 10,000 participants will be enrolled at UC San Diego, Eisenhower Medical Center, and Rady Children's Hospital San Diego.

**Study Duration**

Your medical record will only be accessed as needed for the purposes of this study.

Your samples will be retained by UCSD indefinitely or until the samples are “used up”. You can stop your participation at any time. However, if you decide to stop your participation in the study, please talk to your study doctor first.

If you decide to withdraw, any samples that remain, which can be identified as yours, will be destroyed. However, if the samples have already been used, provided to a researcher for specific use, or de-identified (no code or link to identify the sample as yours), the samples will not be able to be destroyed.

**CONFLICT OF INTEREST**

The Principal Investigator, Dr. Kurzrock, is an unpaid consultant of a collaborating company, Sequenom.

Dr. Kurzrock owns equity/stocks in IDbyDNA and some samples in this study are being sent to IDbyDNA for research.

We are informing you of this information because it may affect your decision to participate in this study.

**PROCEDURES**

If you agree to participate in this study, Dr. Kurzrock and her study team will collect information from your medical record about your general medical history, any information about your tumor “profile”, treatments you receive and how you respond to those treatments. In some cases there may be information in your medical record (chart) that may measure what cancer drugs do to your body (pharmacodynamics) and what your body does to absorb and get rid of the cancer drug from your system (pharmacokinetics). We will collect this information. The researchers estimate that your medical record will be accessed approximately one to four times a year based on your treatment schedule.

No therapy is included in this study. This study will observe the outcomes of how your doctor chooses to treat your cancer. If you have a tumor “profile” researchers will look at whether or not your doctor has treated you with drugs that are matched to genetic abnormalities that may have been found in your profile. This study will also collect information about why your physician chose certain treatments if this information is available in your medical record.

If you are having a procedure called a bronchoalveolar lavage (BAL) as part of routine care, extra BAL fluid may be sent off for genetic analysis. A BAL involves rinsing of the lungs and then a collection of the fluid from the rinsing.

If you are coughing, you may be asked to cough into a collection cup so that testing may be done on the sputum.

Stored or discarded biological samples taken during your routine care that would normally be disposed of and not saved will be analyzed.

The existing specimens collected for this study will be used to examine the levels of cells, protein, RNA (the coding material for protein), and other molecules to see what kind of cells, proteins and/or chemicals are found in the sample. Some studies on the genetic makeup of your cells (DNA is the genetic material that will be studied) may also be done to help find out more about changes in genes that can cause cancer and other diseases. We may also send samples to our collaborators, which may include for-profit companies.

In particular, the study may involve Whole Genome Sequencing (WGS) from existing specimens (for example tissue or blood previously collected), which is a type of genetic testing that examines a person’s DNA to determine if any changes exist that can cause disease. Your tissue, body cavity fluid, blood or urine samples contain genes, which are made up of DNA and which serve as the “instruction book” for the cells that make up our bodies. Your entire unique genetic material, made up of DNA, is known as a genome. A gene is a small segment of DNA that controls the production of a single protein. The WGS studies are intended to look through the DNA for changes that can cause disease. Currently, researchers and doctors know some of the genetic changes that can cause disease, but not all of them. Because WGS examines a larger portion of the DNA than traditional tests, it might discover the cause of disease in cases where other tests did not. WGS may also reveal information about unexpected diseases. Your samples and medical information will help us study how genes interact with other factors to influence the development of diseases such as cancer, cardiovascular disease, diabetes and glaucoma.

Study Biospecimens (such as blood, tissue, or saliva) collected from you for this study and/or information obtained from your biospecimens may be used in this research or other research, and shared with other organizations. You will not share in any commercial value or profit derived from the use of your biospecimens and/or information obtained from them. If you decide later that you do not want the specimens collected from you to be used for future research, you may tell this to Dr. Kurzrock, who will use her best efforts to stop any additional studies. However, in some cases, such as if your samples have already been tested, the data from these tests are no longer linked to your identity and cannot be removed from the research database.

Dr. Kurzrock, her associates, or her successors in these studies will keep your DNA specimen and/or the information derived from it for up to indefinitely.

There will be no direct benefit to you from this study since you will not be provided with any results or information regarding your DNA test. The investigator, however, may learn more about cancer and cancer-related conditions.

Even though we will do our best to keep your information confidential, there is a possibility that your genetic risk for certain diseases is accidentally divulged to the wrong source; if that happens you might be discriminated against in obtaining life or health insurance, employment or the ability to adopt children. The risks associated with *Genetic Studies* and *Loss of Confidentiality* are described above for the main study.

Incidental Findings

In general, we will not give you any individual results from the study of the samples you give us. This is because it will probably take a long time for this project to produce health-related information that we will know how to interpret accurately. It is possible, however, that studies of your samples may reveal an important genetic finding that could have an impact on your healthcare. Such findings are considered incidental, as they are not part of the planned research. In some cases incidental findings may inform you or your doctor about a medical condition that may be treatable. However, it is important to know that these incidental findings were obtained using investigational procedures and are not considered clinically valid. For this reason, steps will be taken when researchers identify through investigational procedures an incidental finding that may be important to your ongoing or future healthcare.

These are the steps that will be followed:

If the incidental finding found through investigational procedures is deemed by your doctor to be significant or important to your healthcare and is actionable based on recommendations from established authorities in the relevant medical field (such as the American College of Medical Genetics and Genomics (ACMG), your doctor may contact you to discuss the finding. You will be asked to provide another sample, which will be sent to a licensed clinical laboratory for confirmation. However, incidental findings that are performed by investigational procedures may not be reported back to you even if they could be important.

If the incidental finding is deemed by your doctor not to be significant or have meaningful impact on your healthcare, you will not be notified of the finding.

A description of the optional procedures involved in this research study and a place for you to note your consent or refusal to participate in these procedures can be found toward the end of this document.

The information collected about you on this study may be kept indefinitely or until the end of this research project. If you decide later that you do not want the information collected about you to be used for future research, you may tell this to Dr. Kurzrock, who will use her best efforts to stop any additional studies. You may also ask to have your data removed from the study database if it has not already been analyzed.

**RISKS OF PARTICIPATION**

Participation in this study may involve some added risks or discomforts. There will be the following risks while you are on this study. You should discuss these with your doctor. There may also be other risks that we cannot predict.

*Risks of Blood Draws:* There is a risk of discomfort or pain, bleeding, swelling and a small arm bruise and swelling when blood is drawn. Rarely, a clot or infection may occur at the site of the blood draw. Some people also become faint, dizzy, or light-headed during or immediately after the blood draw.

*Risks of Loss of Confidential Information:* There is also a small risk that information from your health records will be released to an unauthorized party. We will do our best to make sure that your personal information will be kept private.  The chance that this information will be given to someone else is very small. An identification code assigned by the study team to each patient will be used in place of your name to protect your identity when reporting trial-related information.

*Risks associated with DNA/Genetic Studies:* Instances are known in which a participant in research has been required to furnish genetic information as a precondition for in applying for health insurance and/or a job. Participation in this study does not mean that you have had genetic testing. Genetic testing means having a test performed and the results provided to you and your doctor. If you are interested in having genetic testing performed you should consult your doctor, as some commercial tests are available. Your doctor can provide you with the necessary information to determine if such a test would be appropriate for you.

Although your genetic information is unique to you, you do share some genetic information with your children, parents, brothers, sisters, and other blood relatives. Consequently, it may be possible that genetic information from them could be used to help identify you. Similarly, it may be possible that genetic information from you could be used to help identify them.

Some people involved in genetic studies have felt anxious about the possibility of carrying an altered gene that they could possibly pass on to their children.  Even though we will do our best to keep your information confidential, there is the possibility that your genetic risk for certain diseases is accidently divulged to the wrong source, if that happens you might be discriminated against obtaining life or health insurance, employment or ability to adopt children.

Since some genetic variations can help to predict the future health problems of you and your relatives, this information might be of interest to health providers, life insurance companies, and others. Instances are known in which a patient has been required to give genetic information as a precondition for application for health insurance and/or a job. There are state and federal laws that protect against genetic discrimination.

Federal and State laws generally make it illegal for health insurance companies, group health plans, and most employers to discriminate against you based on your genetic information. This law generally will protect you in the following ways:

a) Health insurance companies and group health plans may not request your genetic information that we get from this research.

b) Health insurance companies and group health plans may not use your genetic information when making decisions regarding your eligibility or premiums.

c) Employers with 5 or more employees may not use your genetic information that we get from this research when making a decision to hire, promote, or fire you or when setting the terms of your employment.

Be aware that these laws **do not** protect you against genetic discrimination by companies that sell life insurance, disability insurance, or long-term care insurance.

**BENEFITS OF PARTICIPATION**

If you agree to take part in this study, there will not be direct medical benefit to you. This study may benefit future patients by advancing knowledge about cancer and genetic profiles to help improve treatments.

**ALTERNATIVES TO PARTICIPATION**

The alternative to participating in this study is to not participate.

**COSTS/COMPENSATION**

There will be no cost to you for participating in this study. There will be no payment to you for participating in this study.

**COMPENSATION FOR RESEARCH-RELATED INJURY**

If you are injured as a direct result of participation in this research, the University of California will provide any medical care you need to treat those injuries. The University will not provide any other form of compensation to you if you are injured. You may call the Human Research Protections Program Office at 858-246-HRPP (858-246-4777) for more information about this, to inquire about your rights as a research subject or to report research-related problems.

**VOLUNTARY PARTICIPATION**

Participation in this study is entirely voluntary. If you choose not to participate or wish to withdraw your consent to participate in these study procedures at any time, it will in no way affect your regular treatments or medical care at this institution or loss of benefits to which you are entitled.

You will be told if any important new information is found during the course of this study that may affect your wanting to continue.

**DO YOU HAVE ANY QUESTIONS?**

Dr. Kurzrock and/or ______________________________ has explained this study to you, and answered your questions. You may contact Dr. Kurzrock at (858) 246-1102. If you have other questions or research-related problems, you may call the Moores UCSD Cancer Center Clinical Trials Office at (858) 822-5354.

If you have questions about your rights as a research participant, your participation in this study, and/or concerns about this study, you may call the UCSD Human Research Protections Program (a group of people who review the research study to protect your rights and welfare) at 858-246-HRPP (858-246-4777).

A description of this clinical trial will be available on www.ClinicalTrials.gov, as required by U.S. Law. This Web site will not include information that can identify you. At most, the Web site will include a summary of the results. You can search this Web site at any time.

**CONFIDENTIALITY**

The confidentiality of your research records will be maintained to the extent permitted by law. This includes using locked filing cabinets and the use of passwords will be required to access your personal data on computers.  Access to your information will be limited to study personnel who need to use it for the purpose of the research in this study. Only the minimum necessary information required will be collected, stored, used and reported. Your medical information will not be made publicly available unless disclosure is required by law or regulation.

The research use may include sharing of your samples with for-profit companies attempting to understand health and disease, and to make drugs to treat cancer and other diseases, and growing your specimen outside of the body. To be useful for research and discovery, the subset of information from your medical record discussed above will also be shared with these companies. Information may also be shared with other researchers at not-for-profit and other academic institutions.  At no time will your identity be shared with non-UCSD researchers.

Information from analyses of your samples and the subset of your medical information will be put into databases along with information from the other research participants. These databases may be accessible by institutions and companies that are studying various diseases. Please note that traditionally-used identifying information about you, such as your name, address, telephone number, or social security number, will NOT be put into the database(s).

Information obtained from this study may be published or given to the UCSD Institutional Review Board and other regulatory agencies such as the Food and Drug Administration (FDA) and the Department of Health and Human Services (DHHS). Your identity will remain confidential.

Study information and samples will be labeled with a code instead of your name or other personal information that can easily identify you.

You will be asked to sign a separate HIPAA authorization form to allow the study team to access and share information from your medical record. Your permission as described in this informed consent and HIPAA document does not have an automatic expiration date.

**OPTIONAL PROCEDURES – Samples for Future Research**

Researchers are interested in the way that genes affect how your body responds to cancer treatment. Genes carry information about features that are found in you and in people who are related to you. Much of this research is done using samples from your tumor tissue, blood, or urine. Through these studies, researchers hope to find new ways to prevent, detect, or treat cancer.

If you choose to take part in this optional study future research tests may be performed on your tissue, blood, urine, or other biological samples that may be collected as a part of this research study, has already been collected as part of your regular care, or that you agree(d) to have collected for the UCSD Moores Cancer Center Biorepository (a separate study that collects and stores samples for cancer research).

Blood: If you choose to allow us to have a sample of your blood, a qualified person will take up to 100 mL of blood from you (about 7 tablespoons). You may also be asked to donate additional small amounts of blood in the future. You will not be allowed to give more than 100 mL (about 7 tablespoons) in an 8-week period. The blood will either be drawn from a catheter if you have one already in place, or by a needle. The sample of blood for research can be drawn at the same time as blood is being taken for routine tests in order to avoid multiple needle sticks.

Urine and Discarded Samples: Biological samples taken during your routine care that would normally be disposed of such as urine or ascites fluid may also be collected for future testing. If you consent to optional future urine collection, a small amount of urine will be collected in a plastic cup at home. If collected at home, a shipping label will be provided to you for its return.

Swabs: A nasal swab may be collected. A nasal swab consists of a long Q-tip being insert into a nostril and brushing the interior of the nose. This brushing can be momentarily uncomfortable and may cause your eyes to tear up or your nose to run. A swab may also be taken of your throat and can cause a gagging reflex. Swabs of your skin, the inside of your cheek, and other swabs may be taken for future research. These swabs are non-invasive meaning they do not break the skin.

Due to rapidly changing technology, the type of testing that will be performed on your samples is yet undetermined; however it will involve genetic research related to cancer. Testing may also involve looking at what cancer drugs are doing to your body (pharmacodynamics) and what your body does to absorb and get rid of the cancer drug from your system (pharmacokinetics).

Each sample will be labeled with a code so that any lab personnel testing the samples will not know your identity. These research tests can involve looking at the genetic make-up of your cells (DNA is the genetic material that will be studies), proteins and RNA (the coding material for protein), and other chemicals that could help understand more about cancer.

We may also send samples to our collaborators, which may include for-profit companies.

Study Biospecimens (such as blood, tissue, or saliva) collected from you for this study and/or information obtained from your biospecimens may be used in this research or other research, and shared with other organizations. You will not share in any commercial value or profit derived from the use of your biospecimens and/or information obtained from them.

Even though we will do our best to keep your information confidential, there is a possibility that your genetic risk for certain diseases is accidentally divulged to the wrong source; if that happens you might be discriminated against in obtaining life or health insurance, employment or the ability to adopt children. The risks associated with *Genetic Studies* and *Loss of Confidentiality* are described above for the main study.

The chance that you will benefit from taking part in this optional procedure is very small. There are no costs to you or your insurance. You will not be paid for taking part.

The information collected about you and results of any tests performed on this study may be kept indefinitely or until the end of this research project.

- If you decide later that you do not want the specimens collected from you to be used for genetic research, you may tell this to Dr Kurzrock and she will use her best efforts to stop additional specimen analysis and/or destroy the specimens if that is what you want. However, in some cases, if the genetic testing has already occurred it might not be possible to remove the results.
- If you decide later that you do not want the information collected about you to be used for future research, you may tell this to Dr. Kurzrock, who will use her best efforts to stop any additional studies. You may also ask to have your data removed from the study database if it has not already been analyzed.

**Consent for Optional Procedures**

You agree to have your existing specimens used for future laboratory studies as described above:

YES_____ NO_____

You agree to have blood collected and used for future laboratory studies as described above:

YES_____ NO_____

You agree to have urine collected and used for future laboratory studies as described above:

YES_____ NO_____

You agree to have samples collected from swabs and used for future laboratory studies as described above:

YES_____ NO_____

_________________

Signature of Participant Date

**OPTIONAL PROCEDURES – Return of Results**

Samples collected as part of this study will have genetic testing as described earlier in this document.

You should not expect to get personal results from research tests done through this study, except for the situation described below. Researchers will study samples and information from many people and it could take many years before they know if the results have any meaning.

However, in some cases, clinically approved tests (Clinical Laboratory Improvement Amendments or CLIA certification) may be performed that have results about inheriting a risk of getting cancer. Tests that have been certified by CLIA have met the federal standards for laboratory testing performed on humans. You need to decide if you want your cancer doctor and/or a genetic counselor to discuss the results with you:

You agree that your cancer doctor and/or genetic counselor can discuss the results of clinically approved tests with you that would allow you to learn about inherited risks of getting cancer:

YES_____ NO_____

_________________

Signature of Participant Date

**MAIN SIGNATURE AND CONSENT**

Your participation in this study is voluntary, and you may refuse to participate or withdraw from the study at any time without prejudice or loss of benefits to which you are otherwise entitled. You will receive a signed copy of this consent document and a copy of “The Experimental Subject’s Bill of Rights” to keep.

You agree to participate.

________________________________

Printed Name of Participant

_________________

Signature of Participant Date

_________________________________

Printed Name of Person Obtaining Consent

__________________

Signature of Person Obtaining Consent Date

Human Research Protections Program University of California, San Diego

(858) 246-HRPP (858-246-4777) 9500 Gilman Drive, Mail Code 0052

(858) 246-3329 (FAX) La Jolla, CA 92093-0052

**EXPERIMENTAL SUBJECT’S BILL OF RIGHTS**

The faculty and staff of the University of California, San Diego wish you to know:

Any person who is requested to consent to participate as a subject in a research study involving a medical experiment, or who is requested to consent on behalf of another, has the right to:

1. Be informed of the nature and purpose of the experiment.
2. Be given an explanation of the procedures to be followed in the medical experiment, and any drug or device to be used.
3. Be given a description of any attendant discomforts and risks reasonably to be expected from the experiment.
4. Be given an explanation of any benefits to the subject reasonably to be expected from the experiment, if applicable.
5. Be given a disclosure of any appropriate alternative procedures, drugs, or devices that might be advantageous to the subject, and their relative risks and benefits.
6. Be informed of the avenues of medical treatment, if any, available to the subject after the experiment if complications should arise.
7. Be given an opportunity to ask any questions concerning the experiment or the procedures involved.
8. Be instructed that consent to participate in the medical experiment may be withdrawn at any time, and the subject may discontinue participation in the medical experiment without prejudice.
9. Be given a copy of a signed and dated written consent form when one is required.
10. Be given the opportunity to decide to consent or not to consent to a medical experiment without the intervention of any element of force, fraud, deceit, duress, coercion, or undue influence on the subject’s decision.

If you have questions regarding a research study, the researcher or his/her assistant will be glad to answer them. You may seek information from the Human Research Protections Program - established for the protection of volunteers in research projects - by calling 858-246-HRPP (858-246-4777) from 7:30 AM to 4:00 PM, Monday through Friday, or by writing to the above address.
